# Associations Between Satisfaction With Aging and Health and Well-being Outcomes Among Older US Adults

**DOI:** 10.1001/jamanetworkopen.2021.47797

**Published:** 2022-02-09

**Authors:** Julia S. Nakamura, Joanna H. Hong, Jacqui Smith, William J. Chopik, Ying Chen, Tyler J. VanderWeele, Eric S. Kim

**Affiliations:** 1 Department of Psychology, University of British Columbia, Vancouver, British Columbia, Canada; 2 Human Flourishing Program, Institute for Quantitative Social Science, Harvard University, Cambridge, Massachusetts; 3 Department of Psychology, University of Michigan, Ann Arbor; 4 Department of Psychology, Michigan State University, East Lansing; 5 Department of Epidemiology, Harvard T.H. Chan School of Public Health, Boston, Massachusetts; 6 Department of Biostatistics, Harvard T.H. Chan School of Public Health, Boston, Massachusetts; 7 Lee Kum Sheung Center for Health and Happiness, Harvard T.H. Chan School of Public Health, Boston, Massachusetts

## Abstract

**Importance:**

Researchers and policy makers are expanding the focus from risk factors of disease to seek potentially modifiable health factors that enhance people’s health and well-being. Understanding if and to what degree aging satisfaction (one’s beliefs about their own aging) is associated with a range of health and well-being outcomes aligns with the interests of older adults, researchers, health systems, and politicians.

**Objectives:**

To evaluate associations between changes in aging satisfaction and 35 subsequent health and well-being outcomes.

**Design, Setting, and Participants:**

This cohort study used data from the Health and Retirement Study, a national, diverse, and longitudinal sample of 13 752 US adults older than 50 years, to evaluate if changes in aging satisfaction (between combined cohorts from 2008 and 2010 and 4 years later, in 2012 and 2014) were subsequently associated with 35 indicators of physical, behavioral, and psychosocial health and well-being in 2016 and 2018. Statistical analysis was conducted from July 24, 2020, to November 6, 2021.

**Exposure:**

Aging satisfaction.

**Main Outcomes and Measures:**

A total of 35 physical (eg, stroke), behavioral (eg, sleep problems), and psychosocial (eg, depression) outcomes were evaluated using multiple linear and generalized linear regression models. Data from all participants, irrespective of how their levels of aging satisfaction changed from the prebaseline to baseline waves, were incorporated into the overall estimate, which was conditional on prior satisfaction.

**Results:**

During the 4-year follow-up period, participants (N = 13 752; 8120 women [59%]; mean [SD] age, 65 [10] years; median age, 64 years [IQR, 56-72 years]; 7507 of 11 824 married [64%]) in the highest (vs lowest) quartile of aging satisfaction had improved physical health (eg, 43% reduced risk of mortality [risk ratio, 0.57; 95% CI, 0.46-0.71]), better health behaviors (eg, 23% increased likelihood of frequent physical activity [risk ratio, 1.23; 95% CI, 1.12-1.34]), and improved psychosocial well-being (eg, higher positive affect [β = 0.51; 95% CI, 0.44-0.58] and lower loneliness [β = −0.41; 95% CI, −0.48 to −0.33]), conditional on prebaseline aging satisfaction.

**Conclusions and Relevance:**

This study suggests that higher aging satisfaction is associated with improved subsequent health and well-being. These findings highlight potential outcomes if scalable aging satisfaction interventions were developed and deployed at scale; they also inform the efforts of policy makers and interventionists who aim to enhance specific health and well-being outcomes. Aging satisfaction may be an important target for future interventions aiming to improve later-life health and well-being.

## Key Points

**Question:**

Is aging satisfaction (one’s beliefs about their own aging) associated with physical, behavioral, and psychosocial outcomes?

**Findings:**

In this nationwide cohort study of US adults older than 50 years, being in the highest (vs lowest) quartile of aging satisfaction was associated with improvements in some health behaviors (eg, increased likelihood of engaging in frequent physical activity), physical health conditions (eg, reduced risk of mortality), and psychosocial well-being factors (eg, reduced risk of depression) 4 years later, conditional on prebaseline aging satisfaction.

**Meaning:**

This study suggests that higher aging satisfaction is associated with improved subsequent health and well-being and highlights potential outcomes if scalable aging satisfaction interventions were developed and deployed at scale.

## Teaser

This cohort study uses data from the Health and Retirement Study to evaluate associations between changes in aging satisfaction and 35 subsequent health and well-being outcomes.

## Introduction

Several prominent intergovernmental organizations (eg, World Health Organization) are urging countries to go “Beyond GDP (gross domestic product),” and use well-being indicators (eg, life satisfaction) in addition to traditional economic indicators when making important policy decisions. Many countries are adopting this paradigm shift.^1,2^ As populations age, identifying factors that foster health and well-being is critical for stemming the growing wave of chronic conditions and mounting health care costs.^3,4^ Researchers and policy makers are increasingly shifting from focusing on risk factors of disease to potentially modifiable health assets that enhance people’s health and well-being.^3,4,5,6,7^ One health asset of increasing interest to older adults, researchers, and health care systems (who seek enhanced health and well-being in our rapidly aging population) is aging satisfaction.

Aging satisfaction refers to self-reported beliefs that people have about their own aging^8^ (ie, quality of life, energy, happiness, and feelings of usefulness)^9^; aging satisfaction has been linked with better physical health (eg, reduced risk of physical functioning limitations, cognitive impairment, and mortality),^8,10,11,12,13,14,15^ health behaviors (eg, increased use of preventive health services, increased medication adherence, and better diet),^16,17,18,19,20,21^ psychological well-being (eg, higher life satisfaction and fewer depressive symptoms),^22,23^ and social outcomes (eg, higher social involvement, perceived support availability, and numbers of new friends).^24,25^ According to stereotype embodiment theory (which states that age stereotypes are internalized, activated, and then work through multiple pathways to influence people’s health and well-being),^26^ aging satisfaction might be associated with physical health via psychological pathways (eg, if people believe poor health is inevitable with age, self-fulfilling prophecies prevent them from engaging in healthy behaviors),^27,28^ physiologic pathways (eg, exposure to negative stereotypes heightens cardiovascular stress responses),^29^ and behavioral pathways (eg, poorer sleep quality and decreased use of preventive health care services).^21,30^

Past studies on this topic have broken new ground in observing associations between aging satisfaction and health and well-being outcomes, but remain somewhat limited in terms of potential utility for interventionists and policy makers. For example, many prior studies evaluated 1 outcome or a limited range of outcomes, rather than evaluating a large number of outcomes simultaneously. Assessing many outcomes within the same study allows us to directly compare the effect sizes of multiple outcomes, which allows interventionists and policy makers to better understand specific changes to health and well-being outcomes that might be observed if interventionists and policy makers intervened on aging satisfaction. Furthermore, almost no longitudinal studies have adjusted for aging satisfaction in the prebaseline wave, which allows researchers to ask a slightly different question: what health and well-being outcomes might we observe within a relatively short time horizon (4-year follow-up) if aging satisfaction was increased?

We evaluated a 4-year follow-up period for several reasons. First, a practical reason, most of our outcomes were assessed every 4 years. Second, many election cycles in the US (as well as in other nations) occur approximately every 4 years; thus, the 4-year time frame represents a reasonable window of time in which a policy maker has to make positive change to be reelected. Our study suggests the health and well-being outcomes that we might expect to observe 4 years later if effective aging satisfaction interventions and policies (prior work suggests that aging satisfaction can be intervened on^31^) were implemented. Third, 4 years is a reasonable amount of time in which aging satisfaction could affect a variety of health and well-being outcomes.

We used the new outcome-wide analytic approach (see Statistical Analysis)^32^ to examine if changes in aging satisfaction were associated with better subsequent health and well-being across physical, behavioral, and psychosocial factors. These outcomes were chosen because they are frequently included in the conceptualization of seminal gerontologic models that characterize the antecedents, processes, and outcomes that foster aging well.^33,34,35,36,37^

## Methods

### Study Population

Participants were from the Health and Retirement Study (HRS), a national sample of US adults older than 50 years. Approximately 50% of HRS respondents were randomly selected for an enhanced face-to-face interview in 2008 when the aging satisfaction measure was first implemented. The other half of respondents were assessed in 2010. After the interview, participants completed a psychosocial questionnaire (response rates were 84% in 2008 and 73% in 2010).^38^ These subcohorts report psychosocial factors every 4 years. Data from 2008 and 2010 were combined to increase the sample size and statistical power; the 2 cohorts were comparable on all study measures. We restricted the sample to people who completed the psychosocial data at baseline because more than half the study outcomes were included in this assessment, resulting in a final sample of 13 752 participants. We used publicly available, deidentified data from the HRS, and were therefore exempt from additional review by the institutional review board at the University of British Columbia. This study followed the Strengthening the Reporting of Observational Studies in Epidemiology (STROBE) reporting guideline.

This study used data from 3 time points. Covariates were assessed in the prebaseline wave (2008 and 2010), the exposure was assessed in the baseline wave (2012 and 2014), and outcomes were assessed in the outcome wave (2016 and 2018). Further study documentation can be found on the HRS website.^39^

### Measures

#### Aging Satisfaction

Aging satisfaction was measured in the baseline wave (2012 and 2014) using an 8-item scale. Five items came from the Attitude Toward Own Aging subscale of the Philadelphia Geriatric Center Morale Scale^9,40^ and 3 others came from the Berlin Aging Study.^41^ Participants answered questions such as, “So far, I am satisfied with the way that I am aging,” on a 6-point Likert scale (where 1 indicates strongly disagree and 6 indicates strongly agree). Negatively worded items were reverse coded and all items were averaged to create an overall score (higher values indicated more positive aging satisfaction; Cronbach α = 0.81). For complete-case analyses, the final score was set to missing if more than 4 items had missing values. To examine potential threshold effects, we created quartiles of the exposure based on the baseline distribution of aging satisfaction.

#### Covariates

We adjusted for a wide range of covariates in the prebaseline wave (2008 and 2010), including: age (continuous), sex (male or female), self-reported race and ethnicity (White, African-American, Hispanic, and other [“other” category taken from a data set with no further breakdown available]), marital status (married or not married), income (<$50 000, $50 000-$74 999, $75 000-$99 999, and ≥$100 000), total wealth (based on quintiles of the score distribution for total wealth in this sample), educational attainment (no degree, GED [General Educational Development certificate] or high school diploma, or ≥ college degree), employment status (yes or no), health insurance (yes or no), geographical region (Northeast, Midwest, South, or West), religious service attendance (none, <1 time/week, or ≥1 time/week), personality (openness, conscientiousness, extraversion, agreeableness, neuroticism; continuous variables), and childhood abuse (yes or no). To evaluate changes in aging satisfaction (conditional on the past), we also adjusted for aging satisfaction in the prebaseline wave (2008 and 2010) because doing so helps reduce the risk of reverse causation and potential unmeasured confounding.^42^

#### Outcomes

We evaluated 35 outcomes in the outcome wave (2016 and 2018) including: physical health (all-cause mortality, number of chronic conditions, diabetes, hypertension, stroke, cancer, heart disease, lung disease, arthritis, overweight or obesity, physical functioning limitations, cognitive impairment, chronic pain, and self-rated health), health behaviors (heavy drinking, smoking, physical activity, and sleep problems), psychological well-being (positive affect, life satisfaction, optimism, purpose in life, mastery [beliefs about one’s ability or self-efficacy], health mastery, and financial mastery), psychological distress (depression, depressive symptoms, hopelessness, negative affect, and perceived constraints), and social factors (loneliness, living with a spouse or partner, and frequency of contact with children, other family, and friends). eMethods 1 in the Supplement and HRS materials provide further details about these variables.^38,43,44^

### Statistical Analysis

Statistical analysis was conducted from July 24, 2020, to November 6, 2021. We used the outcome-wide analytic approach^32^ to assess whether changes in aging satisfaction between the prebaseline wave and the baseline wave were associated with subsequent health and well-being outcomes in the outcome wave. This approach uses several analytic decisions not yet widely used outside of causal inference in biostatistics; thus, we summarize its key features here. First, if covariates are assessed at the same time point as the exposure (baseline wave), it remains unknown if the covariates are confounders or mediators.^32^ Thus, we adjusted for a rich set of covariates in the prebaseline wave to reduce this concern and also help address confounding. Second, we adjusted for all outcomes in the prebaseline wave to reduce risk of reverse causality. Third, to evaluate changes in aging satisfaction, we adjusted for aging satisfaction in the prebaseline wave. This helps hold constant prebaseline aging satisfaction. Data from all participants, irrespective of how their levels of aging satisfaction changed from the prebaseline to baseline waves, were incorporated into the overall estimate. For example, those who were in the highest aging satisfaction quartile in the prebaseline wave and remained there in the baseline wave contributed to the final estimate. However, the estimate produced from this analysis also corresponds to those who were in the lowest aging satisfaction quartile at prebaseline and moved to the highest aging satisfaction quartile at baseline. The model assumes that the highest aging satisfaction quartile coefficient is constant across past aging satisfaction levels (ie, no interaction between past and current aging satisfaction). Thus, we can evaluate how changes in aging satisfaction between the prebaseline wave and the baseline wave are associated with later health and well-being in the outcome wave (eMethods 2 in the Supplement). Adjusting for prebaseline aging satisfaction has other advantages: (1) it reduces the risk of reverse causality by removing the potential accumulating association that aging satisfaction might have had with health and well-being in the past (prevalent exposure) and (2) it allows us to focus on how short-term changes in aging satisfaction (incident exposure) are associated with short-term changes in health and well-being outcomes.

We ran separate models for each outcome, using different models depending on the nature of the outcome: (1) logistic regression for each binary outcome with a prevalence less than 10%, (2) generalized linear models (with a log link and Poisson distribution) for each binary outcome with a prevalence of 10% or more, and (3) linear regression for each continuous outcome.^45,46,47^ We standardized all continuous outcomes (mean = 0 and SD = 1) so their effect sizes can be interpreted as an SD change in the outcome variable. We marked multiple *P* value cutoffs in our tables (including Bonferroni correction) and 95% CIs, because practices for multiple testing vary widely and are continuously evolving.^48,49^ Analyses were conducted in STATA, version 16.1 (StataCorp LLC).

#### Additional Analyses

We conducted several additional analyses, including: (1) E-value analyses to assess the minimum strength that unmeasured confounding must have on the risk ratio scale (with both the exposure and the outcome) to explain away the association between the exposure and outcome, and to evaluate the robustness of our results to potential unmeasured confounding^50^; (2) reanalysis of all models using a reduced list of conventional covariates (eg, sociodemographic factors); (3) reanalysis of all models after removing people with history of a given physical condition at baseline; (4) reanalysis of all models using only complete cases to assess the influence of multiple imputation on results; (5) reanalysis of all models using only the 5 items from the Philadelphia Geriatric Center Morale Scale; (6) reanalysis of all models among only participants displaying increasing aging satisfaction or (7) decreasing aging satisfaction between the prebaseline wave and the baseline wave (the exposure was a difference score between the prebaseline wave and the baseline wave); and (8) reanalysis of all models among only participants displaying minimal changes in aging satisfaction (no movement between quartiles).

#### Multiple Imputation

All missing exposures, covariates, and outcomes were imputed using imputation by chained equations, and 5 data sets were created. This method provides a more flexible approach than other methods of handling missing data^51^ and addresses problems that arise from attrition.^52,53^

## Results

At prebaseline, the mean (SD) age of the 13 752 participants was 65 (10) years (median age, 64 years [IQR, 56-72 years]), and participants were predominantly women (8120 [59%]) and married (7507 of 11 824 [64%]). Table 1 provides the distribution of covariates by quartiles of aging satisfaction. eTable 1 in the Supplement describes changes in aging satisfaction from the prebaseline wave to the baseline wave.

**Table 1.** Characteristics of Participants at Prebaseline by Categories of Aging Satisfaction^a^^,^^b^^,^^c^

Participant characteristics	Aging satisfaction, No. (%)			
	Quartile 1 (n = 2654)	Quartile 2 (n = 2918)	Quartile 3 (n = 2587)	Quartile 4 (n = 2316)
Sociodemographic factors				
Age (range, 48-96 y), median (IQR), y	68.5 (60-75)	68.0 (60-75)	67.0 (59-73)	65.0 (58-71)
Sex				
Female	1649/2654 (62.1)	1698/2918 (58.2)	1556/2587 (60.2)	1388/2316 (59.9)
Male	1005/2654 (37.9)	1220/2918 (41.8)	1031/2587 (39.9)	928/2316 (40.1)
Race and ethnicity				
Black	359/2653 (13.5)	392/2918 (13.4)	353/2586 (13.7)	287/2315 (12.4)
Hispanic	295/2653 (11.1)	277/2918 (9.5)	196/2586 (7.6)	161/2315 (7.0)
White	1927/2653 (72.6)	2170/2918 (74.4)	1985/2586 (76.8)	1815/2315 (78.4)
Other^d^	72/2653 (2.7)	79/2918 (2.7)	52/2586 (2.0)	52/2315 (2.3)
Married	1526/2654 (57.5)	1824/2917 (62.5)	1760/2587 (68.0)	1610/2314 (69.6)
Annual household income				
<$50 000	1876/2654 (70.7)	1719/2918 (58.9)	1270/2587 (49.1)	1002/2316 (43.3)
$50 000-$74 999	358/2654 (13.5)	510/2918 (17.5)	439/2587 (17.0)	401/2316 (17.3)
$75 000-$99 999	181/2654 (6.8)	255/2918 (8.7)	304/2587 (11.8)	283/2316 (12.2)
≥$100 000	239/2654 (9.0)	434/2918 (14.9)	574/2587 (22.2)	630/2316 (27.2)
Total wealth, quintile				
1st	703/2654 (26.5)	560/2918 (19.2)	377/2587 (14.6)	297/2316 (12.8)
2nd	612/2654 (23.1)	640/2918 (21.9)	465/2587 (18.0)	326/2316 (14.1)
3rd	525/2654 (19.8)	599/2918 (20.5)	546/2587 (21.1)	452/2316 (19.5)
4th	492/2654 (18.5)	554/2918 (19.0)	575/2587 (22.2)	544/2316 (23.5)
5th	322/2654 (12.1)	565/2918 (19.4)	624/2587 (24.1)	697/2316 (30.1)
Educational level				
<High school	568/2650 (21.4)	529/2909 (18.2)	320/2580 (12.4)	202/2308 (8.8)
High school	1562/2650 (58.9)	1624/2909 (55.8)	1411/2580 (54.7)	1199/2308 (52.0)
≥College	520/2650 (19.6)	756/2909 (25.0)	849/2580 (32.9)	907/2308 (39.3)
Employed	809/2654 (30.5)	1177/2916 (40.4)	1226/2587 (47.4)	1204/2316 (52.0)
Health insurance	2455/2650 (92.6)	2700/2917 (92.6)	2469/2585 (95.5)	2183/2315 (94.3)
Geographical region				
Northeast	372/2648 (14.1)	414/2916 (14.2)	389/2584 (15.1)	338/2315 (14.6)
Midwest	693/2648 (26.2)	790/2916 (27.1)	646/2584 (25.0)	621/2315 (26.8)
South	1089/2648 (41.1)	1181/2916 (40.5)	1004/2584 (38.9)	863/2315 (37.3)
West	494/2648 (18.7)	531/2916 (18.2)	545/2584 (21.9)	493/2315 (21.3)
Childhood abuse	261/2612 (10.0)	199/2882 (6.9)	184/2565 (7.2)	156/2303 (6.8)
Physical health				
Diabetes	709/2651 (26.7)	636/2914 (21.8)	412/2583 (16.0)	267/2315 (11.5)
Hypertension	1717/2650 (64.8)	1711/2916 (58.7)	1382/2585 (53.5)	1042/2314 (45.0)
Stroke	242/2650 (9.1)	184/2916 (6.3)	145/2585 (5.6)	52/2313 (2.3)
Cancer	414/2646 (15.7)	404/2911 (13.9)	351/2581 (13.6)	262/2315 (11.3)
Heart disease	786/2651 (29.7)	640/2914 (22.0)	454/2585 (17.6)	304/2313 (13.1)
Lung disease	349/2652 (13.2)	255/2916 (8.7)	148/2584 (5.7)	84/2314 (3.6)
Arthritis	1880/2653 (70.9)	1790/2916 (61.4)	1415/2580 (54.8)	989/2312 (42.8)
Overweight or obesity	2029/2620 (77.4)	2168/2877 (75.4)	1835/2561 (71.7)	1513/2297 (65.9)
Physical functioning limitations	1129/2654 (42.5)	575/2918 (19.7)	257/2587 (9.9)	103/2316 (4.5)
Cognitive impairment	514/2606 (19.7)	486/2888 (16.8)	291/2563 (11.4)	199/2297 (8.7)
Chronic pain	1486/2653 (56.0)	1089/2917 (37.3)	710/2586 (27.5)	371/2315 (16.0)
Self-rated health (range, 1-5), mean (SD)	2.6 (1.0)	3.2 (0.9)	3.5 (0.9)	3.9 (0.8)
Health behaviors				
Heavy drinking	134/2157 (6.2)	184/2371 (7.8)	196/2088 (9.4)	162/1877 (8.6)
Smoking	427/2625 (16.3)	361/2904 (12.4)	274/2573 (10.7)	246/2304 (10.7)
Frequent physical activity	1575/2651 (59.4)	2139/2914 (73.4)	2048/2585 (79.2)	1995/2315 (86.2)
Sleep problems	820/1481 (55.4)	680/1643 (41.4)	510/1444 (35.3)	343/1261 (27.2)
Religious service attendance				
Never	806/2651 (30.4)	698/2913 (24.0)	555/2586 (21.5)	532/2316 (23.0)
<1 time/wk	858/2651 (32.4)	922/2913 (31.7)	854/2586 (33.0)	731/2316 (31.6)
≥1 time/wk	987/2651 (37.2)	1293/2913 (44.4)	1177/2586 (45.5)	1053/2316 (45.5)
Psychological well-being, mean (SD)				
Positive affect (range, 1-5)	3.0 (0.7)	3.4 (0.7)	3.9 (0.6)	4.2 (0.6)
Life satisfaction (range, 1-7)	3.9 (1.6)	4.9 (1.4)	5.4 (1.3)	5.8 (1.2)
Optimism (range, 1-6)	3.9 (1.0)	4.3 (0.9)	4.8 (0.8)	5.1 (0.8)
Purpose in life (range, 1-6)	4.1 (0.9)	4.6 (0.8)	5.0 (0.8)	5.3 (0.7)
Mastery (range, 1-6)	4.2 (1.1)	4.7 (1.0)	5.0 (0.9)	5.4 (0.9)
Health mastery (range, 1-10)	6.1 (2.6)	7.3 (2.1)	7.9 (1.8)	8.5 (1.5)
Financial mastery (range, 1-10)	6.1 (3.0)	7.1 (2.5)	7.6 (2.2)	8.2 (1.9)
Psychological distress				
Depression	781/2653 (29.4)	345/2918 (11.8)	141/2587 (5.5)	88/2316 (3.8)
Depressive symptoms (range, 0-8), mean (SD)	2.5 (2.4)	1.3 (1.7)	0.7 (1.3)	0.5 (1.0)
Hopelessness (range, 1-6), mean (SD)	3.1 (1.3)	2.5 (1.1)	1.9 (1.0)	1.6 (0.8)
Negative affect (range, 1-5), mean (SD)	2.1 (0.7)	1.8 (0.6)	1.6 (0.5)	1.4 (0.4)
Perceived constraints (range, 1-6), mean (SD)	2.9 (1.2)	2.3 (1.1)	1.8 (0.9)	1.5 (0.8)
Social factors				
Loneliness (range, 1-3), mean (SD)	1.8 (0.6)	1.5 (0.5)	1.3 (0.4)	1.2 (0.4)
Not living with spouse or partner	1003/2544 (39.4)	933/2788 (33.5)	702/2494 (28.2)	592/2245 (26.4)
Contact <1 time/wk				
Children	700/2593 (27.0)	752/2835 (26.5)	618/2533 (24.4)	541/2263 (23.9)
Other family	1222/2611 (46.8)	1297/2865 (45.3)	1140/2566 (44.4)	1030/2294 (44.9)
Friends	1071/2617 (40.9)	989/2882 (34.3)	780/2563 (30.4)	643/2303 (27.9)
Personality, mean (SD)				
Openness (range, 1-4)	2.7 (0.6)	2.9 (0.5)	3.0 (0.5)	3.2 (0.5)
Conscientiousness (range, 1-4)	3.2 (0.5)	3.3 (0.5)	3.5 (0.4)	3.6 (0.4)
Extraversion (range, 1-4)	2.9 (0.6)	3.1 (0.5)	3.3 (0.5)	3.5 (0.5)
Agreeableness (range, 1-4)	3.4 (0.5)	3.5 (0.5)	3.6 (0.5)	3.7 (0.4)
Neuroticism (range, 1-4)	2.3 (0.6)	2.1 (0.6)	1.9 (0.5)	1.7 (0.5)

^a^ This table was created based on nonimputed data.

^b^ All variables in the table were used as covariates and assessed in the prebaseline wave (2008 and 2010).

^c^ The percentages in some sections may not add up to 100% due to rounding.

^d^ “Other” category taken from a data set with no further breakdown available.

During the 4-year follow-up period, participants in the highest (vs lowest) quartile of aging satisfaction had better physical health across several indicators, including all-cause mortality, number of chronic conditions, diabetes, stroke, cancer, heart disease, lung disease, arthritis, physical functioning limitations, cognitive impairment, chronic pain, and self-rated health, conditional on prebaseline aging satisfaction (Table 2). For example, participants in the highest (vs lowest) quartile of aging satisfaction had a 43% reduced risk of all-cause mortality (risk ratio, 0.57; 95% CI, 0.46-0.71) (eMethods 3 in the Supplement), conditional on prebaseline aging satisfaction. There was less evidence of associations with other physical health outcomes, such as hypertension and overweight or obesity.

**Table 2.** Aging Satisfaction and Subsequent Health and Well-being (Health and Retirement Study)^a^^,^^b^^,^^c^^,^^d^

Outcomes	Aging satisfaction effect estimate, RR, OR, or β (95% CI)			
	Quartile 1 (n = 3468)	Quartile 2 (n = 3942)	Quartile 3 (n = 3226)	Quartile 4 (n = 3116)
Physical health				
All-cause mortality	1 [Reference]	0.72 (0.63 to 0.82)^e^	0.65 (0.55 to 0.77)^e^	0.57 (0.46 to 0.71)^e^
No. of chronic conditions	0 [Reference]	−0.05 (−0.08 to −0.02)^f^	−0.11 (−0.15 to −0.08)^e^	−0.18 (−0.21 to −0.14)^e^
Diabetes	1 [Reference]	0.97 (0.89 to 1.06)	0.94 (0.85 to 1.04)	0.88 (0.78 to 0.99)^g^
Hypertension	1 [Reference]	1.00 (0.95 to 1.06)	0.97 (0.91 to 1.04)	0.94 (0.87 to 1.01)
Stroke	1 [Reference]	0.85 (0.73 to 1.00)^g^	0.79 (0.66 to 0.94)^f^	0.68 (0.54 to 0.84)^e^
Cancer	1 [Reference]	0.94 (0.85 to 1.05)	0.89 (0.78 to 1.00)	0.86 (0.74 to 0.99)^g^
Heart disease	1 [Reference]	0.98 (0.90 to 1.06)	0.92 (0.84 to 1.01)	0.81 (0.72 to 0.90)^e^
Lung disease	1 [Reference]	0.93 (0.83 to 1.04)	0.82 (0.69 to 0.96)^g^	0.74 (0.60 to 0.91)^f^
Arthritis	1 [Reference]	1.00 (0.94 to 1.06)	0.96 (0.89 to 1.02)	0.92 (0.85 to 0.99)^g^
Overweight or obesity	1 [Reference]	1.01 (0.95 to 1.07)	1.02 (0.95 to 1.10)	1.01 (0.94 to 1.09)
Physical functioning limitations	1 [Reference]	0.84 (0.77 to 0.91)^e^	0.68 (0.61 to 0.75)^e^	0.53 (0.44 to 0.64)^e^
Cognitive impairment	1 [Reference]	0.92 (0.83 to 1.03)	0.88 (0.76 to 1.01)	0.83 (0.70 to 0.98)^g^
Chronic pain	1 [Reference]	0.95 (0.88 to 1.01)	0.84 (0.77 to 0.91)^e^	0.73 (0.65 to 0.80)^e^
Self-rated health	0 [Reference]	0.19 (0.15 to 0.23)^e^	0.34 (0.28 to 0.39)^e^	0.46 (0.41 to 0.51)^e^
Health behaviors				
Heavy drinking	1 [Reference]	1.15 (0.82 to 1.62)	1.38 (0.95 to 2.00)	1.19 (0.71 to 2.00)
Smoking	1 [Reference]	1.03 (0.87 to 1.21)	1.04 (0.83 to 1.29)	1.04 (0.82 to 1.32)
Frequent physical activity	1 [Reference]	1.13 (1.05 to 1.22)^f^	1.18 (1.09 to 1.28)^e^	1.23 (1.12 to 1.34)^e^
Sleep problems	1 [Reference]	0.90 (0.83 to 0.98)^g^	0.86 (0.78 to 0.95)^f^	0.77 (0.69 to 0.86)^e^
Psychological well-being				
Positive affect	0 [Reference]	0.18 (0.13 to 0.23)^e^	0.35 (0.30 to 0.39)^e^	0.51 (0.44 to 0.58)^e^
Life satisfaction	0 [Reference]	0.19 (0.12 to 0.25)^e^	0.33 (0.27 to 0.39)^e^	0.45 (0.36 to 0.55)^e^
Optimism	0 [Reference]	0.12 (0.07 to 0.17)^e^	0.22 (0.17 to 0.28)^e^	0.33 (0.26 to 0.40)^e^
Purpose in life	0 [Reference]	0.17 (0.11 to 0.22)^e^	0.31 (0.26 to 0.36)^e^	0.46 (0.37 to 0.55)^e^
Mastery	0 [Reference]	0.18 (0.14 to 0.22)^e^	0.32 (0.26 to 0.39)^e^	0.44 (0.34 to 0.54)^e^
Health mastery	0 [Reference]	0.19 (0.12 to 0.26)^e^	0.34 (0.25 to 0.43)^e^	0.44 (0.34 to 0.53)^e^
Financial mastery	0 [Reference]	0.19 (0.14 to 0.23)^e^	0.29 (0.24 to 0.34)^e^	0.37 (0.30 to 0.44)^e^
Psychological distress				
Depression	1 [Reference]	0.77 (0.67 to 0.88)^e^	0.54 (0.45 to 0.66)^e^	0.45 (0.35 to 0.58)^e^
Depressive symptoms	0 [Reference]	−0.20 (−0.24 to −0.16)^e^	−0.31 (−0.37 to −0.26)^e^	−0.35 (−0.42 to −0.28)^e^
Hopelessness	0 [Reference]	−0.16 (−0.21 to −0.12)^e^	−0.27 (−0.32 to −0.22)^e^	−0.36 (−0.42 to −0.30)^e^
Negative affect	0 [Reference]	−0.17 (−0.21 to −0.12)^e^	−0.29 (−0.34 to −0.24)^e^	−0.42 (−0.49 to −0.36)^e^
Perceived constraints	0 [Reference]	−0.17 (−0.23 to −0.12)^e^	−0.30 (−0.39 to −0.22)^e^	−0.42 (−0.49 to −0.35)^e^
Social factors				
Loneliness	0 [Reference]	−0.18 (−0.22 to −0.14)^e^	−0.28 (−0.34 to −0.23)^e^	−0.41 (−0.48 to −0.33)^e^
Not living with a spouse or partner	1 [Reference]	0.92 (0.86 to 0.99)^g^	0.89 (0.81 to 0.99)^g^	0.89 (0.79 to 1.01)
Contact <1 time/wk				
Children	1 [Reference]	1.02 (0.93 to 1.12)	1.00 (0.89 to 1.13)	0.98 (0.85 to 1.13)
Other family	1 [Reference]	0.99 (0.91 to 1.07)	0.97 (0.88 to 1.06)	0.94 (0.85 to 1.05)
Friends	1 [Reference]	1.00 (0.90 to 1.10)	0.95 (0.86 to 1.06)	0.96 (0.83 to 1.11)

Abbreviations: OR, odds ratio; RR, risk ratio.

^a^ If the reference value is 1, the effect estimate is RR, with the exception of the heavy drinking category, which is OR; if the reference value is 0, the effect estimate is β.

^b^ The analytic sample was restricted to those who had participated in the baseline wave (2012 and 2014). Multiple imputation was performed to impute missing data on the exposure, covariates, and outcomes. All models adjusted for sociodemographic characteristics (age, sex, race and ethnicity, marital status, annual household income, total wealth, educational level, employment status, health insurance, and geographical region), prebaseline childhood abuse, prebaseline religious service attendance, prebaseline values of the outcome variables (diabetes, hypertension, stroke, cancer, heart disease, lung disease, arthritis, overweight or obesity, physical functioning limitations, cognitive impairment, chronic pain, self-rated health, heavy drinking, current smoking status, physical activity, sleep problems, positive affect, life satisfaction, optimism, purpose in life, mastery, health mastery, financial mastery, depressive symptoms, hopelessness, negative affect, perceived constraints, loneliness, living with spouse or partner, contact with children <1 time/week, contact with other family <1 time/week, and contact with friends <1 time/week), personality factors (openness, conscientiousness, extraversion, agreeableness, and neuroticism), and the prebaseline value of the exposure (coded in quartiles). These variables were adjusted for in the wave prebaseline to the exposure assessment (2008 and 2010).

^c^ An outcome-wide analytic approach was used, and a separate model for each outcome was run. A different type of model was run depending on the nature of the outcome: (1) for each binary outcome with a prevalence of 10% or more, a generalized linear model (with a log link and Poisson distribution) was used to estimate an RR; (2) for each binary outcome with a prevalence of less than 10%, a logistic regression model was used to estimate an OR; and (3) for each continuous outcome, a linear regression model was used to estimate a β.

^d^ All continuous outcomes were standardized (mean = 0; SD = 1), and β was the standardized effect size.

^e^ *P* < .05 after Bonferroni correction (the *P* value cutoff for Bonferroni correction is *P* = .05/35 and *P* < .001 for outcomes).

^f^ *P* < .01 before Bonferroni correction.

^g^ *P* < .05 before Bonferroni correction.

When considering health behaviors, participants in the highest (vs lowest) quartile of aging satisfaction had a 23% increased likelihood of subsequent engagement in frequent physical activity (risk ratio, 1.23; 95% CI, 1.12-1.34) and 23% reduced risk of sleep problems (risk ratio, 0.77; 95% CI, 0.69-0.86), conditional on prebaseline aging satisfaction (Table 2). However, there was little evidence of associations between aging satisfaction and either heavy drinking or smoking.

For psychosocial factors, participants in the highest (vs lowest) quartile of aging satisfaction, conditional on prior aging satisfaction, had better subsequent outcomes for all psychological well-being indicators (ie, higher positive affect [β = 0.51; 95% CI, 0.44-0.58], life satisfaction [β = 0.45; 95% CI, 0.36-0.55], optimism [β = 0.33; 95% CI, 0.26-0.40], purpose in life [β = 0.46; 95% CI, 0.37-0.55], mastery [β = 0.44; 95% CI, 0.34-0.54], health mastery [β = 0.44; 95% CI, 0.34-0.53], and financial mastery [β = 0.37; 95% CI, 0.30-0.44]) and psychological distress indicators (ie, lower depression [risk ratio, 0.45; 95% CI, 0.35-0.58], depressive symptoms [β = –0.35; 95% CI, –0.42 to –0.28], hopelessness [β = –0.36; 95% CI, –0.42 to –0.30], negative affect [β = –0.42; 95% CI, –0.49 to –0.36], and perceived constraints [β = –0.42; 95% CI, –0.49 to –0.35]), conditional on prebaseline aging satisfaction (Table 2). Participants also had decreased loneliness (β = −0.41; 95% CI, −0.48 to −0.33), conditional on prebaseline aging satisfaction. However, there was little evidence of associations with other social factors (ie, living with a spouse or partner, or contact with children, other family, or friends).

### Additional Analyses

First, E-values suggested that many of the observed associations were moderately robust to unmeasured confounding (Table 3).^50^ For example, for depression, an unmeasured confounder would have to be associated with both aging satisfaction and depression by risk ratios of 3.85 each (above and beyond the covariates already adjusted for) to explain away the association. Furthermore, to shift the 95% CI to include the null, an unmeasured confounder would have to be associated with both aging satisfaction and depression by risk ratios of 2.85. Second, adjustment for conventional covariates (compared with fully adjusted models) and analyses removing anyone with a history of a given physical condition at baseline showed mostly similar, or larger, estimates (eTable 2 in the Supplement). Third, complete-case analyses showed similar results with results from the main imputed analyses (eTable 3 in the Supplement). Fourth, analyses using only the 5 items from the Philadelphia Geriatric Center Morale Scale Aging Satisfaction subscale showed similar results to the full 8-item measure used in our main analyses (eTable 4 in the Supplement). Fifth, when evaluating only the subsets of participants who displayed increasing or decreasing aging satisfaction, there were fewer associations with physical health conditions (eg, there was less evidence of an association with mortality in participants with increasing aging satisfaction) and those who maintained the same aging satisfaction showed the strongest associations with improved subsequent health and well-being (eTables 5, 6, and 7 in the Supplement).

**Table 3.** E-Values for the Associations Between Aging Satisfaction and Subsequent Health and Well-being (N = 13 752)^a^

Outcomes	Effect estimate^b^	CI limit^c^
Physical health		
All-cause mortality	2.88	2.15
No. of chronic conditions	1.63	1.53
Diabetes	1.54	1.09
Hypertension	1.33	1.00
Stroke	2.32	1.65
Cancer	1.61	1.12
Heart disease	1.79	1.45
Lung disease	2.05	1.44
Arthritis	1.40	1.08
Overweight or obesity	1.13	1.00
Physical functioning limitations	3.17	2.52
Cognitive impairment	1.70	1.17
Chronic pain	2.10	1.79
Self-rated health	2.41	2.26
Health behaviors		
Heavy drinking	1.66	1.00
Smoking	1.25	1.00
Frequent physical activity	1.75	1.49
Sleep problems	1.90	1.58
Psychological well-being		
Positive affect	2.57	2.38
Life satisfaction	2.38	2.14
Optimism	2.03	1.86
Purpose in life	2.41	2.18
Mastery	2.34	2.09
Health mastery	2.35	2.11
Financial mastery	2.15	1.97
Psychological distress		
Depression	3.85	2.85
Depressive symptoms	2.10	1.92
Hopelessness	2.12	1.97
Negative affect	2.30	2.12
Constraints	2.29	2.11
Social factors		
Loneliness	2.25	2.06
Not living with a spouse or partner	1.49	1.00
Contact <1 time/wk		
Children	1.16	1.00
Other family	1.31	1.00
Friends	1.25	1.00

^a^ See VanderWeele and Ding^50^ for the formula for calculating E-values.

^b^ The E-values for effect estimates are the minimum strength that unmeasured confounding may have on the risk ratio scale with both the exposure and the outcome to fully explain away the observed association between the exposure and outcome, conditional on the measured covariates.

^c^ The E-values (fourth vs first quartile) for the limit of the 95% CI closest to the null denote the minimum strength that unmeasured confounding may have on the risk ratio scale with both the exposure and the outcome to shift the CI to include the null value, conditional on the measured covariates.

## Discussion

In this large, longitudinal, and national sample of US adults older than 50 years, those in the highest (vs lowest) quartile of aging satisfaction had improved: physical health (eg, reduced risk of stroke), health behaviors (eg, more frequent physical activity), and psychosocial well-being (eg, lower loneliness). However, there was not substantial evidence of associations with other physical health indicators (eg, hypertension), health behaviors (eg, smoking), and social factors (eg, frequency of contact with friends).

Our findings converge with those of other studies that observed that aging satisfaction is associated with some physical (eg, reduced risk of mortality, physical and cognitive functioning problems, higher self-rated health),^8,10,12,15,22,54,55^ behavioral (eg, physical activity, sleep problems)^20,30^ psychological well-being (eg, higher life satisfaction),^23^ and psychological distress (eg, lower depressive symptoms)^22^ factors. Our findings further converge with those of previous studies that did not observe associations with some health behaviors such as smoking.^19^ Conversely, our findings diverge from those of previous studies that observed associations between aging satisfaction and some physical conditions (eg, obesity),^16^ health behaviors (eg, heavy drinking),^19^ and social integration.^24,25^ There are several potential reasons for diverging results. First, prior studies often used different measures to assess the exposure and outcomes (eg, health behavior composite scores rather than individual behaviors).^17^ However, to help evaluate what impact using a different exposure measure (ie, the 5-item Attitude Toward Own Aging subscale of the Philadelphia Geriatric Center Morale Scale) might have, we conducted additional analyses with this scale and results were similar to results using our full 8-item version. Second, we adjusted for an extensive set of covariates in our study, while most other studies used a more limited set of covariates. Third, a 4-year follow-up period may not be long enough for changes in some health outcomes. However, we were unable to use a longer follow-up period because of the data points available. Fourth, we assessed changes in aging satisfaction (prebaseline adjustment for aging satisfaction), rather than aging satisfaction at one time point. Future studies may benefit from assessing important candidate factors (eg, age, socioeconomic status) that might moderate associations between aging satisfaction and health and well-being, mechanistic pathways underlying the aging satisfaction and health and well-being associations with formal mediation methods, and candidate antecedents of aging satisfaction to identify what factors might modify aging satisfaction.^56^

### Limitations

This study has some limitations. First, nearly all physical health outcomes and health behaviors were self-reported, and thus may be susceptible to self-report bias. However, some outcomes were objective (eg, mortality) and many prior studies have observed links between aging satisfaction and objectively measured health outcomes (eg, C-reactive protein, blood pressure).^15,29,55,57^ In addition, study participants were blinded to this study’s hypotheses and reported aging satisfaction before the outcomes were assessed. Second, there is potential for confounding by third variables. However, we addressed this concern by implementing a longitudinal study design, robust covariate adjustment, and E-value analyses. Third, because the aging satisfaction measure was first introduced to HRS in 2008, we were limited to a 4-year follow-up period (shorter than many prior longitudinal studies on aging satisfaction).^15,54^

## Conclusions

As we aim to recover from the COVID-19 pandemic, a comprehensive and multidisciplinary effort is needed to meet the unique needs of our rapidly aging population, including policy and intervention targets that promote aging well via physical, behavioral, and psychosocial health and well-being. Previous studies have documented that aging satisfaction can potentially be improved,^31^ but further work is needed to develop scalable interventions. These findings highlight the outcomes that we might observe if scalable aging satisfaction interventions were developed and deployed at scale; these findings can inform the efforts of policy makers and interventionists who aim to enhance specific health and well-being outcomes (through direct comparisons of effect sizes between outcomes). Thus, the purpose of our study was to draw attention to specific physical, behavioral, and psychosocial factors that might improve as a result of changes in aging satisfaction in older adults. We aimed to explore a more comprehensive pool of outcomes, within the same study, that investigators should further evaluate deeply in future studies. With further work, macro-level policies and individual-level interventions aimed at improving aging satisfaction may have the potential to enhance a wide range of health and well-being outcomes for the rapidly growing population of older adults in the US.

## Notes

Supplement. **eMethods 1.** Assessment of Outcomes **eMethods 2.** Proof Illustrating How Adjusting for Pre-Baseline Levels of Aging Satisfaction Can Help Us Evaluate How “Change” in Aging Satisfaction Is Associated With Subsequent Health and Well-Being Outcomes Over Time **eMethods 3.** Considering Causes of Death **eTable 1.** Change in Aging Satisfaction from the Pre-Baseline Wave (t_0_) to the Baseline Wave (t_1_) **eTable 2.** Aging Satisfaction and Subsequent Health/Well-Being (Adjustment for Conventional/All Covariates: N = 13 752) **eTable 3.** Complete-Case Analyses: Aging Satisfaction and Subsequent Health/Well-Being (N: 5062 to 8575) **eTable 4.** Aging Satisfaction and Subsequent Health/Well-Being (N = 13 752); 5-Item Philadelphia Geriatric Center Morale Scale **eTable 5.** Increase in Aging Satisfaction and Subsequent Health/Well-Being (Health and Retirement Study: N = 5769) **eTable 6.** Decrease in Aging Satisfaction and Subsequent Health/Well-Being (Health and Retirement Study: N = 7227) **eTable 7.** Stable Aging Satisfaction and Subsequent Health/Well-Being (Health and Retirement Study: N: 4872 to 4881)

## References

[1] Durand M, Exton C. Adopting a well-being approach in central government: policy mechanisms and practical tools. In: Global happiness and wellbeing policy report 2019. Global Council for Happiness and Wellbeing. Accessed September 2, 2021. https://www.teamwork.no/pdf/2019_global-happiness-and-wellbeing.pdf#page=191

[2] Stiglitz JE. Measuring What Counts: The Global Movement for Well-Being. The New Press; 2019.

[3] Kubzansky LD, Huffman JC, Boehm JK, . Positive psychological well-being and cardiovascular health promotion: JACC Health Promotion Series. J Am Coll Cardiol. 2018;72(12):1382-1396. doi:10.1016/j.jacc.2018.07.042 30213332 PMC6289282

[4] VanderWeele TJ, Chen Y, Long K, Kim ES, Trudel-Fitzgerald C, Kubzansky LD. Positive epidemiology? Epidemiology. 2020;31(2):189-193. doi:10.1097/EDE.0000000000001147 31809344

[5] VanderWeele TJ. On the promotion of human flourishing. Proc Natl Acad Sci U S A. 2017;114(31):8148-8156. doi:10.1073/pnas.1702996114 28705870 PMC5547610

[6] Kim ES, Tkatch R, Martin D, MacLeod S, Sandy L, Yeh C. Resilient aging: psychological well-being and social well-being as targets for the promotion of healthy aging. Gerontol Geriatr Med. 2021;7:23337214211002951. doi:10.1177/23337214211002951 33816707 PMC7995285

[7] Kim ES, Chen Y, Nakamura JS, Ryff CD, VanderWeele TJ. Sense of purpose in life and subsequent physical, behavioral, and psychosocial health: an outcome-wide approach. Am J Health Promot. 2022;36(1):137-147. 34405718 10.1177/08901171211038545 PMC8669210

[8] Levy BR, Slade MD, Kasl SV. Longitudinal benefit of positive self-perceptions of aging on functional health. J Gerontol B Psychol Sci Soc Sci. 2002;57(5):409-417. doi:10.1093/geronb/57.5.P409 12198099

[9] Lawton MP. The Philadelphia Geriatric Center Morale Scale: a revision. J Gerontol. 1975;30(1):85-89. doi:10.1093/geronj/30.1.85 1109399

[10] Brown KE, Kim J, Stewart T, Fulton E, McCarrey AC. Positive, but not negative, self-perceptions of aging predict cognitive function among older adults. Int J Aging Hum Dev. 2021;93(1):543-561. doi:10.1177/0091415020917681 32354222

[11] Robertson DA, King-Kallimanis BL, Kenny RA. Negative perceptions of aging predict longitudinal decline in cognitive function. Psychol Aging. 2016;31(1):71-81. doi:10.1037/pag0000061 26691302

[12] Sargent-Cox KA, Anstey KJ, Luszcz MA. The relationship between change in self-perceptions of aging and physical functioning in older adults. Psychol Aging. 2012;27(3):750-760. doi:10.1037/a0027578 22390161

[13] Wurm S, Benyamini Y. Optimism buffers the detrimental effect of negative self-perceptions of ageing on physical and mental health. Psychol Health. 2014;29(7):832-848. doi:10.1080/08870446.2014.891737 24527737

[14] Robertson DA, Savva GM, King-Kallimanis BL, Kenny RA. Negative perceptions of aging and decline in walking speed: a self-fulfilling prophecy. PLoS One. 2015;10(4):e0123260. doi:10.1371/journal.pone.0123260 25923334 PMC4414532

[15] Levy BR, Slade MD, Kunkel SR, Kasl SV. Longevity increased by positive self-perceptions of aging. J Pers Soc Psychol. 2002;83(2):261-270. doi:10.1037/0022-3514.83.2.261 12150226

[16] Levy BR, Slade MD. Positive views of aging reduce risk of developing later-life obesity. Prev Med Rep. 2018;13:196-198. doi:10.1016/j.pmedr.2018.12.012 30705805 PMC6348756

[17] Levy BR, Myers LM. Preventive health behaviors influenced by self-perceptions of aging. Prev Med. 2004;39(3):625-629. doi:10.1016/j.ypmed.2004.02.029 15313104

[18] Klusmann V, Sproesser G, Wolff JK, Renner B. Positive self-perceptions of aging promote healthy eating behavior across the life span via social-cognitive processes. J Gerontol B Psychol Sci Soc Sci. 2019;74(5):735-744. doi:10.1093/geronb/gbx139 29186555

[19] Villiers-Tuthill A, Copley A, McGee H, Morgan K. The relationship of tobacco and alcohol use with ageing self-perceptions in older people in Ireland. BMC Public Health. 2016;16(1):627-627. doi:10.1186/s12889-016-3158-y 27448397 PMC4957865

[20] Wurm S, Tomasik MJ, Tesch-Römer C. On the importance of a positive view on ageing for physical exercise among middle-aged and older adults: cross-sectional and longitudinal findings. Psychol Health. 2010;25(1):25-42. doi:10.1080/08870440802311314 20391205

[21] Kim ES, Moored KD, Giasson HL, Smith J. Satisfaction with aging and use of preventive health services. Prev Med. 2014;69:176-180. doi:10.1016/j.ypmed.2014.09.008 25240763 PMC4424793

[22] Cohn-Schwartz E, Segel-Karpas D, Ayalon L. Longitudinal dyadic effects of aging self-perceptions on health. J Gerontol B Psychol Sci Soc Sci. 2021;76(5):900-909. doi:10.1093/geronb/gbaa082 32572494 PMC8063669

[23] Wurm S, Tomasik MJ, Tesch-Römer C. Serious health events and their impact on changes in subjective health and life satisfaction: the role of age and a positive view on ageing. Eur J Ageing. 2008;5(2):117-127. doi:10.1007/s10433-008-0077-5 28798566 PMC5546269

[24] Menkin JA, Robles TF, Gruenewald TL, Tanner EK, Seeman TE. Positive expectations regarding aging linked to more new friends in later life. J Gerontol B Psychol Sci Soc Sci. 2017;72(5):771-781. 26903172 10.1093/geronb/gbv118 PMC5926985

[25] Schwartz E, Ayalon L, Huxhold O. Exploring the reciprocal associations of perceptions of aging and social involvement. J Gerontol B Psychol Sci Soc Sci. 2021;76(3):563-573. doi:10.1093/geronb/gbaa008 31950185

[26] Levy B. Stereotype embodiment: a psychosocial approach to aging. Curr Dir Psychol Sci. 2009;18(6):332-336. doi:10.1111/j.1467-8721.2009.01662.x 20802838 PMC2927354

[27] Westerhof GJ, Wurm S. Subjective aging and health. In: Braddick O, ed. Oxford Research Encyclopedia of Psychology. Oxford University Press; 2018. doi:10.1093/acrefore/9780190236557.013.4

[28] Wurm S, Warner LM, Ziegelmann JP, Wolff JK, Schüz B. How do negative self-perceptions of aging become a self-fulfilling prophecy? Psychol Aging. 2013;28(4):1088-1097. doi:10.1037/a0032845 24128074

[29] Levy BR, Hausdorff JM, Hencke R, Wei JY. Reducing cardiovascular stress with positive self-stereotypes of aging. J Gerontol B Psychol Sci Soc Sci. 2000;55(4):205-213. doi:10.1093/geronb/55.4.P205 11584877

[30] Lin J-N. Gender differences in self-perceptions about aging and sleep among elderly Chinese residents in Taiwan. J Nurs Res. 2016;24(4):347-356. doi:10.1097/JNR.0000000000000167 27846107

[31] Levy BR, Pilver C, Chung PH, Slade MD. Subliminal strengthening: improving older individuals’ physical function over time with an implicit-age-stereotype intervention. Psychol Sci. 2014;25(12):2127-2135. doi:10.1177/0956797614551970 25326508 PMC4372155

[32] VanderWeele TJ, Mathur MB, Chen Y. Outcome-wide longitudinal designs for causal inference: a new template for empirical studies. Stat Sci. 2020;35(3):437-466. doi:10.1214/19-STS728

[33] Rowe JW, Kahn RL. Human aging: usual and successful. Science. 1987;237(4811):143-149. doi:10.1126/science.3299702 3299702

[34] Ryff CD, Singer B. Understanding healthy aging: key components and their integration. In: Bengston VL, Gans D, Putney NM, and Silverstein M, eds. Handbook of Theories of Aging. 2nd ed. Springer Publishing Co; 2009:117-144.

[35] Reich JW, Zautra AJ, Hall JS, eds. Handbook of Adult Resilience. The Guilford Press; 2010.

[36] Aldwin CM, Igarashi H. Successful, optimal, and resilient aging: a psychosocial perspective. In: Lichtenberg PA, Mast BT, Carpenter BD, Wetherell JL, eds. APA Handbook of Clinical Geropsychology, Vol. 1: History and Status of the Field and Perspectives on Aging. American Psychological Association; 2015:331-359.

[37] Depp CA, Jeste DV. Definitions and predictors of successful aging: a comprehensive review of larger quantitative studies. Am J Geriatr Psychiatry. 2006;14(1):6-20. doi:10.1097/01.JGP.0000192501.03069.bc 16407577

[38] Smith J, Ryan LH, Fisher GG, Sonnega A, Weir DR. HRS psychosocial and lifestyle questionnaire 2006-2016. Health and Retirement Study. Accessed September 2, 2021. https://hrs.isr.umich.edu/publications/biblio/9066

[39] Health and Retirement Study. Accessed December 28, 2021. https://hrsonline.isr.umich.edu/

[40] Liang J, Bollen KA. The structure of the Philadelphia Geriatric Center Morale Scale: a reinterpretation. J Gerontol. 1983;38(2):181-189. doi:10.1093/geronj/38.2.181 6827034

[41] Baltes PB, Mayer KU. The Berlin Aging Study: Aging From 70 to 100. Cambridge University Press; 2001.

[42] VanderWeele TJ, Jackson JW, Li S. Causal inference and longitudinal data: a case study of religion and mental health. Soc Psychiatry Psychiatr Epidemiol. 2016;51(11):1457-1466. doi:10.1007/s00127-016-1281-9 27631394

[43] Fisher GG, Faul JD, Weir DR, Wallace RB. Documentation of chronic disease measures in the Health and Retirement Study. Health and Retirement Study. Accessed September 2, 2021. https://hrs.isr.umich.edu/publications/biblio/5619

[44] Jenkins KR, Ofstedal MB, Weir D. Documentation of health behaviors and risk factors measured in the Health and Retirement Study (HRS/AHEAD). Health and Retirement Study. Accessed September 2, 2021. https://hrs.isr.umich.edu/publications/biblio/5736

[45] VanderWeele TJ. On a square-root transformation of the odds ratio for a common outcome. Epidemiology. 2017;28(6):e58-e60. doi:10.1097/EDE.0000000000000733 28816709 PMC5617805

[46] Zou G. A modified Poisson regression approach to prospective studies with binary data. Am J Epidemiol. 2004;159(7):702-706. doi:10.1093/aje/kwh090 15033648

[47] Austin PC, Laupacis A. A tutorial on methods to estimating clinically and policy-meaningful measures of treatment effects in prospective observational studies: a review. Int J Biostat. 2011;7(1):6. doi:10.2202/1557-4679.1285 22848188 PMC3404554

[48] Dunn OJ. Multiple comparisons among means. J Am Stat Assoc. 1961;56(293):52-64. doi:10.1080/01621459.1961.10482090

[49] VanderWeele TJ, Mathur MB. Some desirable properties of the Bonferroni correction: is the Bonferroni correction really so bad? Am J Epidemiol. 2019;188(3):617-618. doi:10.1093/aje/kwy250 30452538 PMC6395159

[50] VanderWeele TJ, Ding P. Sensitivity analysis in observational research: introducing the E-value. Ann Intern Med. 2017;167(4):268-274. doi:10.7326/M16-2607 28693043

[51] Groenwold RHH, Donders ART, Roes KCB, Harrell FE Jr, Moons KGM. Dealing with missing outcome data in randomized trials and observational studies. Am J Epidemiol. 2012;175(3):210-217. doi:10.1093/aje/kwr302 22262640

[52] Asendorpf JB, Schoot R van de, Denissen JJA, Hutteman R. Reducing bias due to systematic attrition in longitudinal studies: the benefits of multiple imputation. Int J Behav Dev. 2014;38(5):453-460. doi:10.1177/0165025414542713

[53] Harel O, Mitchell EM, Perkins NJ, . Multiple imputation for incomplete data in epidemiologic studies. Am J Epidemiol. 2018;187(3):576-584. doi:10.1093/aje/kwx349 29165547 PMC5860387

[54] Kotter-Grühn D, Kleinspehn-Ammerlahn A, Gerstorf D, Smith J. Self-perceptions of aging predict mortality and change with approaching death: 16-year longitudinal results from the Berlin Aging Study. Psychol Aging. 2009;24(3):654-667. doi:10.1037/a0016510 19739922

[55] Levy BR, Myers LM. Relationship between respiratory mortality and self-perceptions of aging. Psychol Health. 2005;20(5):553-564. doi:10.1080/14768320500066381

[56] Schönstein A, Dallmeier D, Denkinger M, . Health and subjective views on aging: longitudinal findings from the ActiFE Ulm Study. J Gerontol B Psychol Sci Soc Sci. 2021;76(7):1349-1359. doi:10.1093/geronb/gbab023 33528511 PMC8363042

[57] Levy BR, Bavishi A. Survival advantage mechanism: inflammation as a mediator of positive self-perceptions of aging on longevity. J Gerontol B Psychol Sci Soc Sci. 2018;73(3):409-412. 27032428 10.1093/geronb/gbw035 PMC5927092

