# Supplementary material for: Associations Between Satisfaction With Aging and Health and Well-being Outcomes Among Older US Adults
Source: JAMA Netw Open. 2022 Feb 9;5(2):e2147797. doi: 10.1001/jamanetworkopen.2021.47797 (PMC8829664; doi:10.1001/jamanetworkopen.2021.47797)
Supplement: Supplement. — eMethods 1. Assessment of Outcomes eMethods 2. Proof Illustrating How Adjusting for Pre-Baseline Levels of Aging Satisfaction Can Help Us Evaluate How “Change” in Aging Satisfaction Is Associated With Subsequent Health and Well-Being Outcomes Over Time eMethods 3. Considering Causes of Death eTable 1. Change in Aging Satisfaction from the Pre-Baseline Wave (t0) to the Baseline Wave (t1) eTable 2. Aging Satisfaction and Subsequent Health/Well-Being (Adjustment for Conventional/All Covariates: N = 13 752) eTable 3. Complete-Case Analyses: Aging Satisfaction and Subsequent Health/Well-Being (N: 5062 to 8575) eTable 4. Aging Satisfaction and Subsequent Health/Well-Being (N = 13 752); 5-Item Philadelphia Geriatric Center Morale Scale eTable 5. Increase in Aging Satisfaction and Subsequent Health/Well-Being (Health and Retirement Study: N = 5769) eTable 6. Decrease in Aging Satisfaction and Subsequent Health/Well-Being (Health and Retirement Study: N = 7227) eTable 7. Stable Aging Satisfaction and Subsequent Health/Well-Being (Health and Retirement Study: N: 4872 to 4881) [file jamanetwopen-e2147797-s001.pdf]

Supplementary Online Content

Nakamura JS, Hong JH, Smith J, et al. Associations between satisfaction with aging and health and well-being outcomes among older US adults. *JAMA Netw Open.* 2022;5(2):e2147797. doi:10.1001/jamanetworkopen.2021.47797

eMethods 1. Assessment of Outcomes
eMethods 2. Proof Illustrating How Adjusting for Pre-Baseline Levels of Aging Satisfaction Can Help Us Evaluate How "Change" in Aging Satisfaction Is Associated With Subsequent Health and Well-Being Outcomes Over Time
eMethods 3. Considering Causes of Death
eTable 1. Change in Aging Satisfaction from the Pre-Baseline Wave (t_0) to the Baseline Wave (t_1)
eTable 2. Aging Satisfaction and Subsequent Health/Well-Being (Adjustment for Conventional/All Covariates: N = 13 752)
eTable 3. Complete-Case Analyses: Aging Satisfaction and Subsequent Health/Well-Being (N: 5062 to 8575)
eTable 4. Aging Satisfaction and Subsequent Health/Well-Being (N = 13 752); 5-Item Philadelphia Geriatric Center Morale Scale
eTable 5. Increase in Aging Satisfaction and Subsequent Health/Well-Being (Health and Retirement Study: N = 5769)
eTable 6. Decrease in Aging Satisfaction and Subsequent Health/Well-Being (Health and Retirement Study: N = 7227)
eTable 7. Stable Aging Satisfaction and Subsequent Health/Well-Being (Health and Retirement Study: N: 4872 to 4881)

This supplementary material has been provided by the authors to give readers additional information about their work.

© 2022 Nakamura JS et al. *JAMA Network Open.*

eMethods 1. Assessment of Outcomes

Reference Group
The reference group was the healthiest group for all binary outcomes unless otherwise noted.

Physical Health
All-cause mortality. Two methods were used to obtain information about death up to the 2018 questionnaire wave: 1) An exit interview was conducted with next-of-kin. 2) The National Death Index (NDI) was searched for death information after each wave of data collection. There is a 95.5% match when comparing deaths reported by exit interviews versus the NDI.[1]

Chronic conditions. Participants self-reported (yes/no) if they were ever told by a healthcare provider that they had the following conditions: 1) diabetes, 2) hypertension, 3) stroke, 4) cancer, 5) heart disease, 6) lung disease, or 7) arthritis. The HRS has demonstrated validity and reliability of self-reported chronic conditions.[2]

Overweight/obesity. Body mass index (BMI) was derived from self-reported height and weight. It was calculated as weight/height^2 (kg/m^2). A BMI of ≥25 kg/m^2 was considered as overweight/obese.[3]

Number of chronic conditions. To create a score for the number of chronic conditions, a summary score was calculated by summing the number of reported conditions. This measure included the 7 chronic conditions above and also overweight/obesity (range 0-8).

Cognitive functioning limitations. The HRS cognitive functioning assessment[4,5] was adapted from the modified Telephone Interview for Cognitive Status (TICS-M). The assessment included an immediate and delayed 10-noun free recall test, a serial 7 subtraction test, and a backward count 20 test (27-point scale overall). This assessment tool has been shown to have high sensitivity and specificity when assessing cognitive impairment in older adults. The cutpoints used in this study were derived from previous research on cognitive impairment in HRS.[6,7] Participants who scored 0-11 (on the 27-point scale) were classified as having "cognitive impairment", while participants scoring ≥12 were classified as "normal" (the reference group). HRS reports contain further information about these cognitive assessments.[4,5]

Physical functioning limitations. Physical functioning limitations were assessed using items from scales developed by Rosow and Breslau (1966), Nagi (1976), Katz, Ford, Moskowitz, Jackson, and Jaffe (1963), and Lawton and Brody (1969).[8–11] A total of 15 questions about physical functioning (e.g., walking several blocks, climbing one flight of stairs, pushing or pulling large objects, lifting or carrying 10 pounds, getting up from a chair, reaching or extending arms up, stooping, kneeling, or crouching, sitting for 2 hours) and activities of daily living (e.g., walking across a room, dressing, eating, bathing, getting in/out bed, using the toilet, picking up a dime) were included. Participants were classified as having "physical functioning limitations" if they reported >4 limitations with physical functioning, while participants who reported ≤4 limitations were considered "normal" (the reference group). This criterion was determined by identifying the physical function score where 75% of participants could be considered as having healthy physical function at baseline.

© 2022 Nakamura JS et al. *JAMA Network Open.*

Chronic pain. Participants were asked (yes/no): "Are you often troubled with pain?" The reference group was no pain.

Self-rated health. Participants were asked, "Would you say your health is excellent, very good, good, fair, or poor?" on a 5-point scale (reverse coded with higher scores indicating higher self-rated health).

Health Behaviors
Heavy drinking. Following the National Institute on Alcohol Abuse and Alcoholism guidelines,[12] heavy drinking was defined as >14 for drinks/week for men and >7 drinks/week for women. Alcohol consumption was measured by multiplying the number of days/week that alcohol was consumed x number of drinks/day, which resulted in the number of drinks/week. Participants not in this alcohol consumption range were classified as non-heavy drinkers (the reference group).

Smoking. Participants were asked (yes/no): "Do you smoke cigarettes now?" to assess current smoking status. The reference group was "no" smoking.

Frequent physical activity. Based on prior research, a binary physical activity variable was created: ≥1x/week of vigorous or moderate exercise was considered frequent physical activity, while <1x/week of vigorous or moderate exercise was the reference group.[13] Participants indicated the frequency (i.e., response categories: daily, >1x/week, 1x/week, 1-3x/month, hardly ever or never) with which they engaged in vigorous (e.g., running, swimming, aerobics), moderate (e.g., gardening, dancing, walking at a moderate pace), and light (e.g., vacuuming, laundry) activities over the past 12 months.

Sleep problems. Participants completed the 4-item Jenkins Sleep Questionnaire, a widely used and validated screening instrument for assessing sleep complaints and insomnia symptoms.[14] Response categories included "most of the time," "sometimes," and "rarely or never." Healthy sleep (no sleep problems) was defined as reporting "rarely or never" for all four insomnia symptoms assessed (the reference group). People who responded "most of the time" to any of the items were categorized as having sleep problems, and the final results were reverse coded. The sleep questionnaire was only administered every other wave. Thus, sleep data was imputed for half of the sample. Imputed and complete-case analyses showed similar estimates.

Psychological Well-Being
Positive affect. Positive affect was assessed with a 13-item measure based on the Positive and Negative Affect Schedule (PANAS-X).[15] It included the following items: determined, enthusiastic, active, proud, interested, happy, attentive, content, inspired, hopeful, alert, calm, excited, with response categories ranging from 1 (all of the time) to 5 (none of the time). Responses to these 13 items were averaged to obtain an overall score (α=0.92, range 1-5).

Life satisfaction. Life satisfaction was assessed with the 5-item Satisfaction with Life Scale.[16] The scale has shown excellent psychometric properties in prior work. Using a 7-point Likert scale (from 1 (strongly disagree) to 7 (strongly agree)), participants were asked the extent to which they agreed with statements such as, "In most ways my life is close to ideal." Responses

to all items were averaged to create a composite score, with a higher score indicating higher life satisfaction (α=0.88, range 1-7).

Optimism. Optimism was assessed with the Life Orientation Test-Revised (LOT-R), which has good discriminant and convergent validity, as well as good reliability.[17] Using a 6-point Likert scale (from 1 (strongly disagree) to 6 (strongly agree)), participants were asked the degree to which they agreed with statements such as, "In uncertain times, I usually expect the best." Negatively worded items were reverse coded and responses to all items were averaged to create an overall score, with a higher score indicating higher optimism (α=0.75, range 1-6).

Purpose in life. Purpose in life was assessed with a 7-item purpose in life subscale from the Ryff's Psychological Well-Being Scale.[18] The 7-item subscale has been validated in prior work and has shown good psychometric properties.[19] Using a 6-point Likert scale (from 1 (strongly disagree) to 6 (strongly agree)), participants were asked the degree to which they agreed with statements such as, "I have a sense of direction and purpose in my life." Negatively worded items were reverse coded and all items were averaged to create a composite score, with a higher score indicating higher purpose (α=0.75, range 1-6).

Mastery. Mastery was assessed with 5-items derived from Lachman and Weaver (1998).[20] The measure has good discriminant and convergent validity, and good reliability. Using a 6-point Likert scale (from 1 (strongly disagree) to 6 (strongly agree)), participants were asked the degree to which they agreed with statements such as, "I can do just about anything I really set my mind to." All items were averaged to create a composite score, with higher scores indicating higher mastery (α=0.90, range 1-6).

Health mastery. Participants were asked, "How would you rate the amount of control you have over your health these days?" on a 0 ("no control at all") to 10 ("very much control") scale.

Financial mastery. Participants were asked, "How would you rate the amount of control you have over your financial situation these days?" on a 0 ("no control at all") to 10 ("very much control") scale.

Psychological Distress

Depressive symptoms and depression. Depressive symptoms were measured using The Center for Epidemiologic Studies Depression Scale (CESD).[21] Participants indicated the presence of 8 depressive symptoms (e.g., "Much of the time during the past week, I felt depressed") over the past week (yes/no). All items were summed, with a higher score indicating higher depressive symptoms (α=0.80, range 0-8). This scale has been validated in the HRS.[22] Participants with a score of ≥4 were classified as having depression, as done previously (no depression was the reference group).[22] Prior work has suggested that the cutoff value of 4 would produce results similar to the 16-item cutoff when using the full (20-item) CESD scale.[22]

Hopelessness. Hopelessness was assessed with a 4-item questionnaire from two previously validated scales.[23,24] Using a 6-point Likert scale (from 1 (strongly disagree) to 6 (strongly agree)), participants were asked the degree to which they agree with statements such as, "The

© 2022 Nakamura JS et al. *JAMA Network Open.*

future seems hopeless to me and I can't believe that things are changing for the better." All items were averaged to create a composite score (α=0.86, range 1-6).

Negative affect. Negative affect was assessed with a 12-item measure based on the Positive and Negative Affect Schedule (PANAS-X).[15] It included the following items: afraid, upset, guilty, scared, frustrated, bored, hostile, jittery, ashamed, nervous, sad, distressed, with response categories ranging from 1 (all of the time) to 5 (none of the time). Responses to these 12 items were averaged to obtain an overall score (α=0.89, range 1-5).

Perceived constraints. Perceived constraints were assessed with 5 other items derived from Lachman and Weaver (1998),[20] and this measure has good discriminant and convergent validity, as well as good reliability. Using a 6-point Likert scale (from 1 (strongly disagree) to 6 (strongly agree)), participants were asked the degree to which they agreed with statements such as, "What happens in my life is often beyond my control." All items were averaged to create an overall score, with higher scores indicating a higher sense of constraints on personal control (α=0.86, range 1-6).

Social Factors

Loneliness. Loneliness was assessed with three items from the previously validated UCLA Loneliness Scale.[25] Participants answered the following questions: How much of the time do you feel: 1) you lack companionship, 2) left out, and 3) isolated from others, with response categories ranging from 1 (often) to 3 (hardly ever or never). Responses were reverse scored and averaged, with a higher score indicating higher loneliness (α=0.80, range 1-3).

Living with partner/spouse. Participants were asked, "Do you have a husband, wife, or partner with whom you live?", and answered yes/no.

Frequency of Contact with: Children, Other Family, and Friends. Frequency of contact was measured as the frequency with which participants were in contact with their children, other family, or friends (separately). Participants were asked, "On average, how often do you do each of the following?" 1) "Meet up (include both arranged and chance meetings)," 2) "Speak on the phone," 3) "Write or email," and had the choice of the following 6 responses: 1) ≥3x/week, 2) 1x-2x/week, 3) 1-2x/month, 4) every few months, 5) 1-2x/year, 6) <1x/year or never.[26] The highest value on any of the three modes of contact was taken for each relationship type since contact (regardless of the mode of contact) was the main point of interest. For example, if the respondent did not speak on the phone very often with a given person but met them in person very often, contact was operationalized as being common. Two categories of contact were created: 1) frequent contact: ≥1x/week contact (the reference group) and 2) infrequent contact: <1x/week of contact.

Other Factors

Personality. Personality was assessed with 26 items derived from the Midlife Development Inventory Personality scales (MIDI) and International Personality Item Pool (IPIP): the "Big-5" personality traits (openness to experience, conscientiousness, extraversion, agreeableness, and neuroticism),[27] The goal of MIDI was to create the shortest possible measure that assessed the Big-5 personality traits with high validity and reliability using existing trait inventories. In a pilot

© 2022 Nakamura JS et al. *JAMA Network Open.*

study with a probability sample of 1,000 adults aged 30-70, the items with the highest item-to-total correlations and factor loadings were selected for the MIDI. Next, forward regressions were computed to determine the smallest number of items needed to account for more than 90 percent of the total scale variance. For example, items on the conscientiousness scale included "organized," "responsible," "hardworking," and "careless." Response categories ranged from 1 (a lot) to 4 (not at all). Responses were reverse scored so that a higher score indicated higher indication of a given personality trait. All items were averaged to obtain a composite score for each personality trait.

© 2022 Nakamura JS et al. *JAMA Network Open.*

eMethods 2. Proof Illustrating How Adjusting for Pre-Baseline Levels of Aging Satisfaction Can Help Us Evaluate How "Change" in Aging Satisfaction Is Associated With Subsequent Health and Well-Being Outcomes Over Time

Let Y be the outcome in 2016/2018, A_1 the aging satisfaction exposure in 2012/2014, A_0 the aging satisfaction exposure in 2008/2010, C the set of covariates in 2008/2010. For a continuous outcome, the regression model is: $E[Y|a_0, a_1, c] = v + b_0a_0 + b_1a_1 + b_2'c$

Let Y_a denote the potential outcome Y for an individual under an intervention to set A_1 to a. For an individual with baseline aging satisfaction exposure $A_0=a_0$ and covariates c in 2008/2010, under the no-confounding (and positivity and consistency) and modeling assumptions, a change in aging satisfaction of d points $A_0=a_0$ to $A_1=a_0+d$ in 2012/2014, rather than maintaining aging satisfaction of $A_1=a_0$ in 2012/2014, will give rise to an effect (a difference in potential outcomes for Y) of:

$E[Y_{a0+d}| A_0=a_0, c] - E[Y_{a0}| A_0=a_0, c]$
$= E[Y_{a0+d}| A_1=a_0+d, A_0=a_0, c] - E[Y_{a0}| A_1=a_0, A_0=a_0, c]$
$= E[Y| A_1=a_0+d, A_0=a_0, c] - E[Y| A_1=a_0, A_0=a_0, c]$
$= [v + b_0a_0 + b_1(a_0+d) + b_2'c] - [v + b_0a_0 + b_1a_0 + b_2'c]$
$= b_1d$

where the first equality follows by the no-confounding assumption, the second by consistency, and the third by the statistical model.

© 2022 Nakamura JS et al. *JAMA Network Open.*

eMethods 3. Considering Causes of Death

We considered the idea of creating aggregate measures that combined the incidence of a condition and death due to that condition. However, out of the 14 ways HRS categorizes causes of death, very few categories cleanly mapped onto health conditions we evaluated in this study without a large risk of misclassification error. Thus, we did not pursue this option. Causes of death included deaths due to: 1) Musculoskeletal system and connective tissue; 2) Heart, circulatory and blood conditions; 3) Allergies; hay fever; sinusitis; tonsillitis; 4) Endocrine, metabolic and nutritional conditions; 5) Digestive system (stomach, liver, gallbladder, kidney, bladder); 6) Neurological and sensory conditions; 7) Reproductive system and prostate conditions; 8) Emotional and psychological conditions; 9) Miscellaneous; 10) Other symptoms; 11) Not a health condition; 12) None; 13) Other health condition; 14) Cancers and tumors; skin conditions.

© 2022 Nakamura JS et al. *JAMA Network Open.*

eReferences

1. Weir DR. Validating mortality ascertainment in the Health and Retirement Study. Published online 2016. https://hrs.isr.umich.edu/publications/biblio/9022

2. Fisher GG, Faul JD, Weir DR, Wallace RB. Documentation of chronic disease measures in the Heath and Retirement Study (HRS/AHEAD). Published 2005. https://hrs.isr.umich.edu/publications/biblio/5619

3. World Health Organization. Physical status: the use and interpretation of anthropometry: report of a WHO expert committee. Published online 1995. https://apps.who.int/iris/bitstream/handle/10665/37003/WHO_TRS_854.pdf

4. Fisher GG, Halimah H, Faul JD, Rogers WL, Weir DR. Health and Retirement Study imputation of cognitive functioning measures:1992 – 2014. Health and Retirement Study. Published January 13, 2017. https://hrs.isr.umich.edu/sites/default/files/biblio/COGIMPdd.pdf

5. Ofstedal MB, Fisher GG, Herzog AR. Documentation of cognitive functioning measures in the Health and Retirement Study. Published 2005. http://hrsonline.isr.umich.edu/sitedocs/userg/dr-006.pdf

6. Crimmins EM, Kim JK, Langa KM, Weir DR. Assessment of cognition using surveys and neuropsychological assessment: the health and retirement study and the aging, demographics, and memory study. *J Gerontol B Psychol Sci Soc Sci*. 2011;66 Suppl 1:i162-171. doi:10.1093/geronb/gbr048

7. Langa KM, Plassman BL, Wallace RB, et al. The aging, demographics, and memory study: study design and methods. *Neuroepidemiology*. 2005;25(4):181-191. doi:10.1159/000087448

8. Rosow I, Breslau N. A Guttman health scale for the aged. *J Gerontol*. 1966;21(4):556-559. doi: 10.1093/geronj/21.4.556

9. Nagi SZ. An epidemiology of disability among adults in the United States. *Milbank Mem Fund Q Health Soc*. 1976;54(4):439-467. doi: 10.2307/3349677

10. Katz S, Ford AB, Moskowitz RW, Jackson BA, Jaffe MW. Studies of illness in the aged: the index of ADL: a standardized measure of biological and psychosocial function. *JAMA*. 1963;185(12):914-919. doi: 10.1001/jama.1963.03060120024016

11. Lawton MP, Brody EM. Assessment of older people: self-maintaining and instrumental activities of daily living. *The Gerontologist*. 1969;9(3 Part 1):179-186. doi:10.1093/geront/9.3_Part_1.179

12. Drinking levels defined | National Institute on Alcohol Abuse and Alcoholism (NIAAA). Accessed March 31, 2019. https://www.niaaa.nih.gov/alcohol-health/overview-alcohol-consumption/moderate-binge-drinking

© 2022 Nakamura JS et al. *JAMA Network Open.*

13. Nandi A, Glymour MM, Subramanian SV. Association among socioeconomic status, health behaviors, and all-cause mortality in the United States. *Epidemiol Camb Mass*. 2014;25(2):170-177. doi:10.1097/EDE.0000000000000038

14. Jenkins CD, Stanton B-A, Niemcryk SJ, Rose RM. A scale for the estimation of sleep problems in clinical research. *J Clin Epidemiol*. 1988;41(4):313-321. doi:10.1016/0895-4356(88)90138-2

15. Watson D, Clark LA. The PANAS-X: Manual for the positive and negative affect schedule-expanded form. Published online 1994. https://ir.uiowa.edu/cgi/viewcontent.cgi?article=1011&context=psychology_pubs

16. Diener E, Emmons RA, Larsen RJ, Griffin S. The satisfaction with life scale. *J Pers Assess*. 1985;49(1):71-75. doi:10.1207/s15327752jpa4901_13

17. Scheier MF, Carver CS, Bridges MW. Distinguishing optimism from neuroticism (and trait anxiety, self-mastery, and self-esteem): a reevaluation of the Life Orientation Test. *J Pers Soc Psychol*. 1994;67:1063-1078. doi:10.1037/0022-3514.67.6.1063

18. Ryff CD, Keyes CLM. The structure of psychological well-being revisited. *J Pers Soc Psychol*. 1995;69:719-727. doi:10.1037/0022-3514.69.4.719

19. Abbott R, Ploubidis G, Huppert F, Kuh D, Wadsworth M, Croudace T. Psychometric evaluation and predictive validity of Ryff's psychological well-being items in a UK birth cohort sample of women. *Health Qual Life Outcomes*. 2006;4:76. doi:10.1186/1477-7525-4-76

20. Lachman ME, Weaver SL. The sense of control as a moderator of social class differences in health and well-being. *J Pers Soc Psychol*. 1998;74:763-773. doi:10.1037/0022-3514.74.3.763

21. Radloff LS. The CES-D Scale: A self-report depression scale for research in the general population. *Appl Psychol Meas*. 1977;1:385-401. doi:10.1177/014662167700100306

22. Steffeck D. Documentation of affective functioning measures in the Health and Retirement Study. Published online 2000. ttps://hrs.isr.umich.edu/sites/default/files/biblio/dr-005.pdf

23. Beck AT, Weissman A, Lester D, Trexler L. The measurement of pessimism: the hopelessness scale. *J Consult Clin Psychol*. 1974;42:861-865. doi:10.1037/h0037562

24. Everson SA, Kaplan GA, Goldberg DE, Salonen R, Salonen JT. Hopelessness and 4-year progression of carotid atherosclerosis: the Kuopio ischemic heart disease risk factor study. *Arterioscler Thromb Vasc Biol*. 1997;17:1490-1495. doi:10.1161/01.ATV.17.8.1490

25. Russell DW. UCLA Loneliness Scale (Version 3): reliability, validity, and factor structure. *J Pers Assess*. 1996;66(1):20-40. doi:10.1207/s15327752jpa6601_2

© 2022 Nakamura JS et al. *JAMA Network Open.*

26. Teo AR, Choi H, Andrea SB, et al. Does mode of contact with different types of social relationships predict depression in older adults? Evidence from a nationally representative survey. *J Am Geriatr Soc*. 2015;63(10):2014-2022. doi:10.1111/jgs.13667

27. Lachman M, Weaver S. Midlife Development Inventory (MIDI) personality scales: scale construction and scoring (Technical Report). Waltham, MA: Brandeis University, Psychology Department. Published online 1997.

eTable 1. Change in Aging Satisfaction from the Pre-Baseline Wave (t_0) to the Baseline Wave (t_1)[a,b]

**Pre-Baseline Wave (t_0)**	**Baseline Wave (t_1)**			
	Quartile 1	Quartile 2	Quartile 3	Quartile 4
	%	%	%	%
Quartile 1	56.8	29.4	10.1	3.6
Quartile 2	25.7	40.5	23.8	10.0
Quartile 3	12.4	29.4	33.9	24.3
Quartile 4	4.5	14.2	27.3	54.0

[a]The percent of people in quartile 1, 2, 3, or 4 in the pre-baseline wave (t_0) who end up in a quartile 1, 2, 3, or 4 later in the baseline wave (t_1).
[b]The values in the first row (quartile 1) do not add up to 100% because of rounding.

© 2022 Nakamura JS et al. *JAMA Network Open.*

eTable 2. Aging Satisfaction and Subsequent Health/Well-Being (Adjustment for Conventional/All Covariates: N = 13 752)[a,b,c]

**Outcomes**	**Aging Satisfaction**		
	Quartile 1	Conventionally-Adjusted Models[d] Quartile 4	Fully-Adjusted Models[e] Quartile 4
	(Reference)	RR/OR/β (95% CI)	RR/OR/β (95% CI)
**Physical Health**			
All-cause mortality	1.00	0.41 (0.34, 0.49)***	0.57 (0.46, 0.71)***
Number of chronic conditions	0.00	-0.62 (-0.67, -0.57)***	-0.18 (-0.21, -0.14)***
Diabetes[f]	1.00	0.70 (0.54, 0.90)**	0.81 (0.60, 1.08)
Hypertension[g]	1.00	0.74 (0.60, 0.92)**	0.86 (0.66, 1.13)
Stroke[h]	1.00	0.52 (0.36, 0.76)**	0.67 (0.42, 1.06)
Cancer[i]	1.00	0.87 (0.65, 1.16)	0.86 (0.61, 1.22)
Heart disease[j]	1.00	0.57 (0.46, 0.70)***	0.64 (0.50, 0.82)***
Lung disease[k]	1.00	0.51 (0.38, 0.69)***	0.90 (0.59, 1.38)
Arthritis[l]	1.00	0.58 (0.46, 0.72)***	0.75 (0.57, 0.98)*
Overweight/obesity[m]	1.00	0.99 (0.70, 1.40)	1.13 (0.80, 1.60)
Physical functioning limitations[n]	1.00	0.38 (0.30, 0.49)***	0.57 (0.42, 0.78)**
Cognitive impairment[o]	1.00	0.76 (0.63, 0.92)**	0.88 (0.70, 1.11)
Chronic pain[p]	1.00	0.52 (0.45, 0.60)***	0.76 (0.63, 0.90)**
Self-rated health	0.00	0.99 (0.95, 1.04)***	0.46 (0.41, 0.51)***
**Health Behaviors**			
Heavy drinking	1.00	1.07 (0.76, 1.50)	1.19 (0.71, 2.00)*
Smoking	1.00	0.65 (0.55, 0.78)***	1.04 (0.82, 1.32)
Frequent physical activity	1.00	1.45 (1.35, 1.57)***	1.23 (1.12, 1.34)***
Sleep problems	1.00	0.53 (0.49, 0.58)***	0.77 (0.69, 0.86)***
**Psychological Well-Being**			
Positive affect	0.00	1.10 (1.02, 1.18)***	0.51 (0.44, 0.58)***
Life satisfaction	0.00	0.99 (0.93, 1.05)***	0.45 (0.36, 0.55)***
Optimism	0.00	0.94 (0.88, 1.00)***	0.33 (0.26, 0.40)***
Purpose in life	0.00	0.96 (0.89, 1.03)***	0.46 (0.37, 0.55)***
Mastery	0.00	0.86 (0.79, 0.92)***	0.44 (0.34, 0.54)***
Health mastery	0.00	0.89 (0.81, 0.97)***	0.44 (0.34, 0.53)***
Financial mastery	0.00	0.74 (0.69, 0.80)***	0.37 (0.30, 0.44)***

© 2022 Nakamura JS et al. *JAMA Network Open.*

eTable 2. (continued)

Outcomes	Aging Satisfaction		
	Quartile 1	Conventionally-Adjusted Models[d] Quartile 4	Fully-Adjusted Models[e] Quartile 4
	(Reference)	RR/OR/β (95% CI)	RR/OR/β (95% CI)
**Psychological Distress**			
Depression	1.00	0.16 (0.13, 0.20)***	0.45 (0.35, 0.58)***
Depressive symptoms	0.00	-0.90 (-0.96, -0.85)***	-0.35 (-0.42, -0.28)***
Hopelessness	0.00	-0.90 (-0.95, -0.85)***	-0.36 (-0.42, -0.30)***
Negative affect	0.00	-0.94 (-1.00, -0.88)***	-0.42 (-0.49, -0.36)***
Perceived constraints	0.00	-0.92 (-1.00, -0.85)***	-0.42 (-0.49, -0.35)***
**Social Factors**			
Loneliness	0.00	-0.91 (-0.98, -0.84)***	-0.41 (-0.48, -0.33)***
Not living with a spouse/partner	1.00	0.83 (0.76, 0.91)***	0.89 (0.79, 1.01)
Contact children <1x/week	1.00	0.88 (0.80, 0.97)*	0.98 (0.85, 1.13)
Contact other family <1x/week	1.00	0.91 (0.84, 1.00)*	0.94 (0.85, 1.05)
Contact friends <1x/week	1.00	0.81 (0.73, 0.90)***	0.96 (0.83, 1.11)

Note. Abbreviations: CI, confidence interval; OR, odds ratio; RR, risk ratio.
[a]If the reference value is "1," the effect estimate is OR or RR; if the reference value is "0," the effect estimate is β.
[c]An outcome-wide analytic approach was used, and a separate model for each outcome was run. A different type of model was run depending on the nature of the outcome: 1) for each binary outcome with a prevalence of ≥10%, a generalized linear model (with a log link and Poisson distribution) was used to estimate a RR; 2) for each binary outcome with a prevalence of <10%, a logistic regression model was used to estimate an OR; and 3) for each continuous outcome, a linear regression model was used to estimate a β.
[c]All continuous outcomes were standardized (mean=0; standard deviation=1), and β was the standardized effect size.
[d]The analytic sample was restricted to those who had participated in the baseline wave (t_1;2012 or 2014). Multiple imputation was performed to impute missing data on the exposure, covariates, and outcomes. All models adjusted for sociodemographic characteristics (age, sex, race/ethnicity, marital status, annual household income, total wealth, level of education). These variables were adjusted for in the pre-baseline wave (t_0;2008 or 2010).
[e]The analytic sample was restricted to those who had participated in the baseline wave (t_1;2012 or 2014). Multiple imputation was performed to impute missing data on the exposure, covariates, and outcomes. All models adjusted for sociodemographic characteristics (age, sex, race/ethnicity, marital status, annual household income, total wealth, level of education, employment status, health insurance, geographic region), pre-baseline childhood abuse, pre-baseline religious service attendance, pre-baseline values of the outcome variables (diabetes, hypertension, stroke, cancer, heart disease, lung disease, arthritis, overweight/obesity, physical functioning limitations, cognitive impairment, chronic pain, self-rated health, heavy drinking, current smoking status, physical activity, sleep problems, positive affect, life satisfaction, optimism, purpose in life, mastery, health mastery, financial mastery, depressive symptoms, hopelessness, negative affect, perceived constraints, loneliness, living with spouse/partner, contact children

© 2022 Nakamura JS et al. *JAMA Network Open.*

<1x/week, contact other family <1x/week, contact friends <1x/week), personality factors (openness, conscientiousness, extraversion, agreeableness, neuroticism) and the pre-baseline value of the exposure (coded in quartiles). These variables were adjusted for in the wave pre-baseline to the exposure assessment (t_0;2008 or 2010).
[f]Includes only study participants with no history of diabetes (n=10,463).
[g]Includes only study participants with no history of hypertension (n=5,286).
[h]Includes only study participants with no history of stroke (n=12,605).
[i]Includes only study participants with no history of cancer (n=11,529).
[j]Includes only study participants with no history of heart disease (n=10,295).
[k]Includes only study participants with no history of lung disease (n=12,356).
[l]Includes only study participants with no history of arthritis (n=5,375). For this analysis, we did not adjust for arthritis in wave 1 because the cell size was too small and the analysis did not converge. This is the only outcome for which we did not adjust for the outcome in wave 1.
[m]Includes only study participants who were not overweight/obese (n=3,856).
[n]Includes only study participants who did not have physical functioning limitations (n=10,335).
[o]Includes only study participants who did not have cognitive impairment (n=10,983).
[p]Includes only study participants who did not have chronic pain (n=8,593).
*p<0.05 before Bonferroni correction; **p<0.01 before Bonferroni correction; ***p<0.05 after Bonferroni correction (the p-value cutoff for Bonferroni correction is p=0.05/35 outcomes=p<0.001).

© 2022 Nakamura JS et al. *JAMA Network Open.*

eTable 3. Complete-Case Analyses: Aging Satisfaction and Subsequent Health/Well-Being (N: 5062 to 8575)[a,b,c,d]

Outcomes	Aging Satisfaction			
	Quartile 1	Quartile 2	Quartile 3	Quartile 4
	(Reference)	RR/OR/β (95% CI)	RR/OR/β (95% CI)	RR/OR/β (95% CI)
**Physical Health**				
All-cause mortality	1.00	0.68 (0.58, 0.80)***	0.63 (0.51, 0.78)***	0.56 (0.43, 0.73)***
Number of chronic conditions	0.00	-0.03 (-0.07, 0.01)	-0.10 (-0.15, -0.05)***	-0.17 (-0.22, -0.12)***
Diabetes	1.00	0.97 (0.85, 1.10)	0.94 (0.81, 1.09)	0.87 (0.73, 1.03)
Hypertension	1.00	1.01 (0.93, 1.10)	0.97 (0.88, 1.07)	0.93 (0.84, 1.04)
Stroke	1.00	0.90 (0.73, 1.10)	0.81 (0.62, 1.05)	0.70 (0.52, 0.94)*
Cancer	1.00	0.98 (0.84, 1.14)	0.85 (0.71, 1.02)	0.87 (0.71, 1.05)
Heart disease	1.00	0.99 (0.88, 1.11)	0.93 (0.81, 1.07)	0.81 (0.69, 0.96)*
Lung disease	1.00	0.95 (0.79, 1.13)	0.91 (0.73, 1.15)	0.82 (0.62, 1.08)
Arthritis	1.00	0.99 (0.92, 1.08)	0.96 (0.88, 1.06)	0.92 (0.83, 1.03)
Overweight/obesity	1.00	1.02 (0.94, 1.11)	1.04 (0.94, 1.14)	1.03 (0.92, 1.14)
Physical functioning limitations	1.00	0.78 (0.69, 0.89)***	0.68 (0.58, 0.80)***	0.49 (0.40, 0.61)***
Cognitive impairment	1.00	0.94 (0.81, 1.10)	0.87 (0.72, 1.05)	0.89 (0.72, 1.10)
Chronic pain	1.00	0.90 (0.82, 1.00)	0.80 (0.71, 0.91)***	0.70 (0.61, 0.81)***
Self-rated health	0.00	0.24 (0.19, 0.30)***	0.38 (0.32, 0.44)***	0.50 (0.43, 0.57)***
**Health Behaviors**				
Heavy drinking	1.00	1.37 (0.96, 1.95)	1.54 (1.05, 2.26)*	1.15 (0.75, 1.75)
Smoking	1.00	1.04 (0.81, 1.32)	1.06 (0.79, 1.41)	1.01 (0.73, 1.40)
Frequent physical activity	1.00	1.11 (1.01, 1.22)*	1.19 (1.07, 1.32)**	1.21 (1.08, 1.36)***
Sleep problems	1.00	0.87 (0.79, 0.97)*	0.88 (0.77, 0.99)*	0.80 (0.69, 0.93)**
**Psychological Well-Being**				
Positive affect	0.00	0.16 (0.10, 0.22)***	0.36 (0.30, 0.43)***	0.54 (0.47, 0.62)***
Life satisfaction	0.00	0.20 (0.14, 0.27)***	0.33 (0.26, 0.40)***	0.46 (0.38, 0.54)***
Optimism	0.00	0.10 (0.04, 0.16)***	0.26 (0.19, 0.33)***	0.31 (0.24, 0.39)***
Purpose in life	0.00	0.15 (0.09, 0.21)***	0.32 (0.25, 0.38)***	0.45 (0.37, 0.52)***
Mastery	0.00	0.20 (0.13, 0.27)***	0.35 (0.27, 0.43)***	0.44 (0.35, 0.52)***
Health mastery	0.00	0.22 (0.15, 0.29)***	0.39 (0.31, 0.46)***	0.47 (0.39, 0.56)***
Financial mastery	0.00	0.26 (0.19, 0.33)***	0.31 (0.23, 0.38)***	0.36 (0.28, 0.45)***

© 2022 Nakamura JS et al. *JAMA Network Open.*

eTable 3. (continued)

Outcomes	Aging Satisfaction			
	Quartile 1	Quartile 2	Quartile 3	Quartile 4
	(Reference)	RR/OR/β (95% CI)	RR/OR/β (95% CI)	RR/OR/β (95% CI)
**Psychological Distress**				
Depression	1.00	0.69 (0.58, 0.81)***	0.50 (0.40, 0.64)***	0.41 (0.30, 0.56)***
Depressive symptoms	0.00	-0.24 (-0.29, -0.18)***	-0.33 (-0.40, -0.27)***	-0.36 (-0.43, -0.29)***
Hopelessness	0.00	-0.19 (-0.25, -0.12)***	-0.33 (-0.39, -0.26)***	-0.37 (-0.44, -0.29)***
Negative affect	0.00	-0.14 (-0.20, -0.08)***	-0.24 (-0.31, -0.18)***	-0.38 (-0.45, -0.30)***
Perceived constraints	0.00	-0.20 (-0.26, -0.13)***	-0.29 (-0.37, -0.22)***	-0.41 (-0.49, -0.33)***
**Social Factors**				
Loneliness	0.00	-0.19 (-0.26, -0.13)***	-0.28 (-0.36, -0.21)***	-0.40 (-0.48, -0.32)***
Not living with a spouse/partner	1.00	0.96 (0.85, 1.09)	0.87 (0.75, 1.01)	0.86 (0.73, 1.02)
Contact children <1x/week	1.00	1.04 (0.90, 1.21)	1.00 (0.85, 1.19)	0.98 (0.82, 1.18)
Contact other family <1x/week	1.00	1.01 (0.90, 1.13)	0.99 (0.87, 1.13)	0.96 (0.83, 1.10)
Contact friends <1x/week	1.00	0.98 (0.87, 1.11)	0.92 (0.80, 1.06)	0.96 (0.82, 1.13)

Note. Abbreviations: CI, confidence interval; OR, odds ratio; RR, risk ratio.

[a]If the reference value is "1," the effect estimate is OR or RR; if the reference value is "0," the effect estimate is β.

[b]The analytic sample was restricted to those who had participated in the baseline wave (t_1;2012 or 2014). Multiple imputation was performed to impute missing data on the exposure, covariates, and outcomes. All models adjusted for sociodemographic characteristics (age, sex, race/ethnicity, marital status, annual household income, total wealth, level of education, employment status, health insurance, geographic region), pre-baseline childhood abuse, pre-baseline religious service attendance, pre-baseline values of the outcome variables (diabetes, hypertension, stroke, cancer, heart disease, lung disease, arthritis, overweight/obesity, physical functioning limitations, cognitive impairment, chronic pain, self-rated health, heavy drinking, current smoking status, physical activity, sleep problems, positive affect, life satisfaction, optimism, purpose in life, mastery, health mastery, financial mastery, depressive symptoms, hopelessness, negative affect, perceived constraints, loneliness, living with spouse/partner, contact children <1x/week, contact other family <1x/week, contact friends <1x/week), personality factors (openness, conscientiousness, extraversion, agreeableness, neuroticism) and the pre-baseline value of the exposure (coded in quartiles). These variables were adjusted for in the wave pre-baseline to the exposure assessment (t_0;2008 or 2010).

[c]An outcome-wide analytic approach was used, and a separate model for each outcome was run. A different type of model was run depending on the nature of the outcome: 1) for each binary outcome with a prevalence of ≥10%, a generalized linear model (with a log link and Poisson distribution) was used to estimate a RR; 2) for each binary outcome with a prevalence of <10%, a logistic regression model was used to estimate an OR; and 3) for each continuous outcome, a linear regression model was used to estimate a β.

[d]All continuous outcomes were standardized (mean=0; standard deviation=1), and β was the standardized effect size.

*p<0.05 before Bonferroni correction; **p<0.01 before Bonferroni correction; ***p<0.05 after Bonferroni correction (the p-value cutoff for Bonferroni correction is p=0.05/35 outcomes=p<0.001).

© 2022 Nakamura JS et al. *JAMA Network Open.*

eTable 4. Aging Satisfaction and Subsequent Health/Well-Being (N = 13 752); 5-Item Philadelphia Geriatric Center Morale Scale[a,b,c,d]

**Outcomes**	**Aging Satisfaction**			
	Quartile 1 (n=3756)	Quartile 2 (n=3769)	Quartile 3 (n=3176)	Quartile 4 (n=3051)
	(Reference)	RR/OR/β (95% CI)	RR/OR/β (95% CI)	RR/OR/β (95% CI)
**Physical Health**				
All-cause mortality	1.00	0.82 (0.72, 0.94)**	0.76 (0.64, 0.89)***	0.64 (0.52, 0.80)***
Number of chronic conditions	0.00	-0.05 (-0.08, -0.02)***	-0.09 (-0.12, -0.06)***	-0.14 (-0.18, -0.11)***
Diabetes	1.00	0.98 (0.90, 1.07)	0.96 (0.87, 1.06)	0.91 (0.81, 1.03)
Hypertension	1.00	1.00 (0.94, 1.06)	0.97 (0.91, 1.04)	0.94 (0.88, 1.01)
Stroke	1.00	0.86 (0.72, 1.03)	0.86 (0.73, 1.02)	0.73 (0.59, 0.91)**
Cancer	1.00	0.97 (0.88, 1.08)	0.91 (0.80, 1.03)	0.93 (0.81, 1.07)
Heart disease	1.00	0.96 (0.89, 1.04)	0.95 (0.87, 1.04)	0.85 (0.76, 0.95)**
Lung disease	1.00	0.92 (0.82, 1.03)	0.86 (0.74, 1.01)	0.74 (0.61, 0.90)**
Arthritis	1.00	0.99 (0.94, 1.04)	0.96 (0.90, 1.02)	0.93 (0.86, 1.00)
Overweight/obesity	1.00	1.00 (0.94, 1.06)	1.01 (0.95, 1.08)	1.01 (0.94, 1.08)
Physical functioning limitations	1.00	0.82 (0.75, 0.89)***	0.70 (0.63, 0.78)***	0.60 (0.51, 0.69)***
Cognitive impairment	1.00	0.96 (0.87, 1.06)	0.92 (0.80, 1.07)	0.89 (0.75, 1.05)
Chronic pain	1.00	0.92 (0.86, 0.99)*	0.86 (0.79, 0.93)***	0.76 (0.69, 0.85)***
Self-rated health	0.00	0.17 (0.13, 0.21)***	0.29 (0.24, 0.33)***	0.40 (0.35, 0.45)***
**Health Behaviors**				
Heavy drinking	1.00	1.09 (0.78, 1.54)	1.22 (0.77, 1.94)	1.07 (0.60, 1.93)
Smoking	1.00	1.02 (0.88, 1.18)	1.06 (0.83, 1.35)	1.02 (0.82, 1.27)
Frequent physical activity	1.00	1.13 (1.06, 1.22)***	1.17 (1.09, 1.26)***	1.21 (1.11, 1.31)***
Sleep problems	1.00	0.90 (0.84, 0.97)**	0.86 (0.79, 0.95)**	0.82 (0.74, 0.90)***
**Psychological Well-Being**				
Positive affect	0.00	0.19 (0.14, 0.25)***	0.32 (0.27, 0.36)***	0.49 (0.41, 0.58)***
Life satisfaction	0.00	0.20 (0.14, 0.26)***	0.33 (0.27, 0.40)***	0.46 (0.37, 0.56)***
Optimism	0.00	0.11 (0.07, 0.15)***	0.20 (0.15, 0.25)***	0.31 (0.26, 0.36)***
Purpose in life	0.00	0.17 (0.12, 0.23)***	0.27 (0.21, 0.34)***	0.42 (0.31, 0.52)***
Mastery	0.00	0.20 (0.16, 0.24)***	0.32 (0.27, 0.38)***	0.43 (0.36, 0.50)***
Health mastery	0.00	0.15 (0.06, 0.24)**	0.28 (0.20, 0.37)***	0.38 (0.26, 0.50)***
Financial mastery	0.00	0.17 (0.12, 0.21)***	0.24 (0.19, 0.29)***	0.34 (0.27, 0.40)***

© 2022 Nakamura JS et al. *JAMA Network Open.*

eTable 4. (continued)

Outcomes	Aging Satisfaction			
	Quartile 1 (n=3756)	Quartile 2 (n=3769)	Quartile 3 (n=3176)	Quartile 4 (n=3051)
	(Reference)	RR/OR/β (95% CI)	RR/OR/β (95% CI)	RR/OR/β (95% CI)
**Psychological Distress**				
Depression	1.00	0.81 (0.70, 0.93)**	0.56 (0.48, 0.66)***	0.42 (0.32, 0.55)***
Depressive symptoms	0.00	-0.18 (-0.22, -0.14)***	-0.27 (-0.33, -0.22)***	-0.32 (-0.39, -0.26)***
Hopelessness	0.00	-0.14 (-0.19, -0.10)***	-0.22 (-0.28, -0.17)***	-0.33 (-0.37, -0.28)***
Negative affect	0.00	-0.18 (-0.22, -0.14)***	-0.27 (-0.33, -0.22)***	-0.40 (-0.45, -0.35)***
Perceived constraints	0.00	-0.16 (-0.21, -0.12)***	-0.27 (-0.34, -0.20)***	-0.36 (-0.42, -0.31)***
**Social Factors**				
Loneliness	0.00	-0.18 (-0.23, -0.13)***	-0.27 (-0.35, -0.19)***	-0.39 (-0.46, -0.32)***
Not living with a spouse/partner	1.00	0.93 (0.87, 1.00)	0.89 (0.82, 0.97)*	0.90 (0.80, 1.00)
Contact children <1x/week	1.00	1.04 (0.94, 1.15)	1.05 (0.94, 1.18)	1.05 (0.93, 1.19)
Contact other family <1x/week	1.00	1.00 (0.92, 1.07)	0.98 (0.90, 1.07)	0.96 (0.87, 1.07)
Contact friends <1x/week	1.00	0.96 (0.88, 1.05)	0.95 (0.86, 1.04)	0.93 (0.83, 1.05)

Note. Abbreviations: CI, confidence interval; OR, odds ratio; RR, risk ratio.

[a]If the reference value is "1," the effect estimate is OR or RR; if the reference value is "0," the effect estimate is β.

[b]The analytic sample was restricted to those who had participated in the baseline wave (t_1;2012 or 2014). Multiple imputation was performed to impute missing data on the exposure, covariates, and outcomes. All models adjusted for sociodemographic characteristics (age, sex, race/ethnicity, marital status, annual household income, total wealth, level of education, employment status, health insurance, geographic region), pre-baseline childhood abuse, pre-baseline religious service attendance, pre-baseline values of the outcome variables (diabetes, hypertension, stroke, cancer, heart disease, lung disease, arthritis, overweight/obesity, physical functioning limitations, cognitive impairment, chronic pain, self-rated health, heavy drinking, current smoking status, physical activity, sleep problems, positive affect, life satisfaction, optimism, purpose in life, mastery, health mastery, financial mastery, depressive symptoms, hopelessness, negative affect, perceived constraints, loneliness, living with spouse/partner, contact children <1x/week, contact other family <1x/week, contact friends <1x/week), personality factors (openness, conscientiousness, extraversion, agreeableness, neuroticism) and the pre-baseline value of the exposure (coded in quartiles). These variables were adjusted for in the wave pre-baseline to the exposure assessment (t_0;2008 or 2010).

[c]An outcome-wide analytic approach was used, and a separate model for each outcome was run. A different type of model was run depending on the nature of the outcome: 1) for each binary outcome with a prevalence of ≥10%, a generalized linear model (with a log link and Poisson distribution) was used to estimate a RR; 2) for each binary outcome with a prevalence of <10%, a logistic regression model was used to estimate an OR; and 3) for each continuous outcome, a linear regression model was used to estimate a β.

[d]All continuous outcomes were standardized (mean=0; standard deviation=1), and β was the standardized effect size.

© 2022 Nakamura JS et al. *JAMA Network Open.*

*p<0.05 before Bonferroni correction; **p<0.01 before Bonferroni correction; ***p<0.05 after Bonferroni correction (the p-value cutoff for Bonferroni correction is p=0.05/35 outcomes=p<0.001).

© 2022 Nakamura JS et al. *JAMA Network Open.*

eTable 5. Increase in Aging Satisfaction and Subsequent Health/Well-Being (Health and Retirement Study [HRS]: N = 5769)[a,b,c,d]

Outcomes	Aging Satisfaction			
	Quartile 1 (n=1580)	Quartile 2 (n=1291)	Quartile 3 (n=1451)	Quartile 4 (n=1447)
	(Reference)	RR/OR/β (95% CI)	RR/OR/β (95% CI)	RR/OR/β (95% CI)
**Physical Health**				
All-cause mortality	1.00	0.95 (0.72, 1.24)	0.88 (0.68, 1.14)	0.99 (0.75, 1.32)
Number of chronic conditions	0.00	-0.02 (-0.08, 0.03)	-0.03 (-0.08, 0.02)	-0.08 (-0.13, -0.03)**
Diabetes	1.00	1.00 (0.86, 1.17)	1.03 (0.90, 1.19)	0.96 (0.82, 1.11)
Hypertension	1.00	1.01 (0.92, 1.11)	0.99 (0.90, 1.08)	0.97 (0.88, 1.07)
Stroke	1.00	1.01 (0.75, 1.35)	0.92 (0.70, 1.20)	0.92 (0.72, 1.18)
Cancer	1.00	0.96 (0.79, 1.17)	0.91 (0.72, 1.14)	0.86 (0.71, 1.04)
Heart disease	1.00	1.02 (0.88, 1.17)	1.00 (0.87, 1.16)	0.95 (0.82, 1.10)
Lung disease	1.00	1.06 (0.84, 1.34)	1.07 (0.87, 1.32)	0.88 (0.70, 1.11)
Arthritis	1.00	0.96 (0.87, 1.06)	0.97 (0.88, 1.07)	0.97 (0.89, 1.07)
Overweight/obesity	1.00	0.99 (0.90, 1.08)	0.99 (0.90, 1.08)	0.99 (0.90, 1.08)
Physical functioning limitations	1.00	0.90 (0.77, 1.06)	0.90 (0.76, 1.06)	0.81 (0.68, 0.97)*
Cognitive impairment	1.00	1.08 (0.91, 1.27)	1.08 (0.93, 1.26)	1.08 (0.92, 1.26)
Chronic pain	1.00	0.97 (0.85, 1.11)	0.98 (0.87, 1.12)	0.88 (0.76, 1.02)
Self-rated health	0.00	0.04 (-0.04, 0.12)	0.14 (0.08, 0.20)***	0.19 (0.12, 0.26)***
**Health Behaviors**				
Heavy drinking	1.00	1.14 (0.70, 1.84)	1.24 (0.75, 2.05)	1.21 (0.58, 2.51)
Smoking	1.00	0.95 (0.73, 1.24)	0.94 (0.73, 1.22)	1.06 (0.83, 1.35)
Frequent physical activity	1.00	1.04 (0.94, 1.15)	1.06 (0.96, 1.17)	1.09 (0.99, 1.19)
Sleep problems	1.00	0.93 (0.82, 1.06)	0.93 (0.82, 1.05)	0.86 (0.76, 0.97)*
**Psychological Well-Being**				
Positive affect	0.00	0.04 (-0.04, 0.12)	0.10 (0.03, 0.16)**	0.22 (0.14, 0.30)***
Life satisfaction	0.00	0.05 (-0.05, 0.15)	0.12 (0.04, 0.19)**	0.21 (0.13, 0.28)***
Optimism	0.00	0.01 (-0.06, 0.08)	0.05 (-0.03, 0.12)	0.14 (0.07, 0.21)***
Purpose in life	0.00	0.06 (-0.01, 0.13)	0.12 (0.03, 0.20)*	0.22 (0.15, 0.28)***
Mastery	0.00	0.03 (-0.06, 0.12)	0.08 (-0.01, 0.17)	0.20 (0.11, 0.29)***
Health mastery	0.00	0.09 (0.00, 0.18)*	0.12 (0.04, 0.20)**	0.20 (0.11, 0.30)***
Financial mastery	0.00	0.07 (-0.02, 0.15)	0.08 (0.00, 0.15)*	0.14 (0.04, 0.25)**

© 2022 Nakamura JS et al. *JAMA Network Open.*

eTable 5. (continued)

Outcomes	Aging Satisfaction			
	Quartile 1 (n=1580)	Quartile 2 (n=1291)	Quartile 3 (n=1451)	Quartile 4 (n=1447)
	(Reference)	RR/OR/β (95% CI)	RR/OR/β (95% CI)	RR/OR/β (95% CI)
**Psychological Distress**				
Depression	1.00	0.84 (0.63, 1.12)	0.72 (0.58, 0.89)**	0.68 (0.55, 0.85)***
Depressive symptoms	0.00	-0.08 (-0.16, -0.01)*	-0.12 (-0.18, -0.06)***	-0.19 (-0.27, -0.11)***
Hopelessness	0.00	-0.04 (-0.11, 0.03)	-0.06 (-0.14, 0.03)	-0.16 (-0.23, -0.09)***
Negative affect	0.00	-0.08 (-0.17, 0.02)	-0.10 (-0.17, -0.04)**	-0.21 (-0.29, -0.14)***
Perceived constraints	0.00	-0.06 (-0.14, 0.01)	-0.08 (-0.18, 0.02)	-0.19 (-0.26, -0.12)***
**Social Factors**				
Loneliness	0.00	-0.02 (-0.09, 0.05)	-0.08 (-0.15, -0.01)*	-0.19 (-0.27, -0.11)***
Not living with a spouse/partner	1.00	1.05 (0.92, 1.20)	1.00 (0.88, 1.14)	1.01 (0.90, 1.13)
Contact children <1x/week	1.00	1.06 (0.91, 1.24)	1.06 (0.91, 1.22)	1.02 (0.88, 1.17)
Contact other family <1x/week	1.00	0.99 (0.87, 1.12)	1.00 (0.89, 1.11)	0.95 (0.84, 1.08)
Contact friends <1x/week	1.00	0.94 (0.79, 1.12)	0.96 (0.84, 1.11)	0.99 (0.83, 1.19)

Note. Abbreviations: CI, confidence interval; OR, odds ratio; RR, risk ratio.

[a]If the reference value is "1," the effect estimate is OR or RR; if the reference value is "0," the effect estimate is β.

[b]The analytic sample was restricted to those who had participated in the baseline wave (t_1;2012 or 2014) and had an increase in aging satisfaction between the pre-baseline and baseline wave. Quartile 4 shows the estimates for participants with the largest increases in aging satisfaction from the pre-baseline to baseline waves, and other quartiles can be interpreted in the same way. Multiple imputation was performed to impute missing data on the exposure, covariates, and outcomes. All models adjusted for sociodemographic characteristics (age, sex, race/ethnicity, marital status, annual household income, total wealth, level of education, employment status, health insurance, geographic region), pre-baseline childhood abuse, pre-baseline religious service attendance, pre-baseline values of the outcome variables (diabetes, hypertension, stroke, cancer, heart disease, lung disease, arthritis, overweight/obesity, physical functioning limitations, cognitive impairment, chronic pain, self-rated health, heavy drinking, current smoking status, physical activity, sleep problems, positive affect, life satisfaction, optimism, purpose in life, mastery, health mastery, financial mastery, depressive symptoms, hopelessness, negative affect, perceived constraints, loneliness, living with spouse/partner, contact children <1x/week, contact other family <1x/week, contact friends <1x/week), personality factors (openness, conscientiousness, extraversion, agreeableness, neuroticism) and the pre-baseline value of the exposure (coded in quartiles). These variables were adjusted for in the wave pre-baseline to the exposure assessment (t_0;2008 or 2010).

[c]An outcome-wide analytic approach was used, and a separate model for each outcome was run. A different type of model was run depending on the nature of the outcome: 1) for each binary outcome with a prevalence of ≥10%, a generalized linear model (with a log link and Poisson distribution) was

© 2022 Nakamura JS et al. *JAMA Network Open.*

used to estimate a RR; 2) for each binary outcome with a prevalence of <10%, a logistic regression model was used to estimate an OR; and 3) for each continuous outcome, a linear regression model was used to estimate a β.
[d]All continuous outcomes were standardized (mean=0; standard deviation=1), and β was the standardized effect size.
*p<0.05 before Bonferroni correction; **p<0.01 before Bonferroni correction; ***p<0.05 after Bonferroni correction (the p-value cutoff for Bonferroni correction is p=0.05/35 outcomes=p<0.001).

© 2022 Nakamura JS et al. *JAMA Network Open.*

eTable 6. Decrease in Aging Satisfaction and Subsequent Health/Well-Being (Health and Retirement Study [HRS]: N = 7227)[a,b,c,d]

Outcomes	Aging Satisfaction			
	Quartile 1 (n=1814)	Quartile 2 (n=2051)	Quartile 3 (n=1545)	Quartile 4 (n=1817)
	(Reference)	RR/OR/β (95% CI)	RR/OR/β (95% CI)	RR/OR/β (95% CI)
**Physical Health**				
All-cause mortality	1.00	1.14 (0.93, 1.39)	1.10 (0.85, 1.43)	1.47 (1.19, 1.82)***
Number of chronic conditions	0.00	0.03 (-0.02, 0.07)	0.07 (0.02, 0.12)**	0.10 (0.05, 0.15)***
Diabetes	1.00	1.10 (0.97, 1.25)	1.10 (0.97, 1.26)	1.10 (0.96, 1.26)
Hypertension	1.00	1.03 (0.95, 1.11)	1.02 (0.93, 1.10)	1.03 (0.95, 1.12)
Stroke	1.00	1.02 (0.80, 1.28)	1.15 (0.91, 1.45)	1.30 (1.04, 1.61)*
Cancer	1.00	1.00 (0.86, 1.15)	1.07 (0.92, 1.26)	1.12 (0.96, 1.31)
Heart disease	1.00	1.02 (0.91, 1.14)	1.04 (0.92, 1.17)	1.10 (0.97, 1.24)
Lung disease	1.00	1.03 (0.84, 1.25)	1.06 (0.82, 1.36)	1.18 (0.98, 1.42)
Arthritis	1.00	0.99 (0.92, 1.08)	1.02 (0.94, 1.11)	1.02 (0.94, 1.11)
Overweight/obesity	1.00	0.99 (0.91, 1.07)	1.01 (0.93, 1.10)	0.99 (0.91, 1.08)
Physical functioning limitations	1.00	1.08 (0.95, 1.22)	1.14 (1.00, 1.31)	1.39 (1.20, 1.60)***
Cognitive impairment	1.00	1.05 (0.92, 1.20)	1.04 (0.88, 1.22)	1.23 (1.04, 1.46)*
Chronic pain	1.00	1.03 (0.93, 1.14)	1.07 (0.96, 1.20)	1.12 (1.00, 1.24)*
Self-rated health	0.00	-0.07 (-0.15, 0.02)	-0.14 (-0.24, -0.05)**	-0.28 (-0.34, -0.22)***
**Health Behaviors**				
Heavy drinking	1.00	0.83 (0.56, 1.22)	0.86 (0.55, 1.36)	0.78 (0.55, 1.10)
Smoking	1.00	0.94 (0.73, 1.21)	0.92 (0.69, 1.21)	0.90 (0.73, 1.10)
Frequent physical activity	1.00	0.98 (0.90, 1.07)	0.94 (0.86, 1.03)	0.89 (0.81, 0.98)*
Sleep problems	1.00	1.00 (0.90, 1.11)	1.07 (0.96, 1.19)	1.17 (1.03, 1.33)*
**Psychological Well-Being**				
Positive affect	0.00	-0.04 (-0.11, 0.02)	-0.11 (-0.19, -0.03)**	-0.26 (-0.32, -0.19)***
Life satisfaction	0.00	-0.06 (-0.13, 0.02)	-0.14 (-0.21, -0.07)***	-0.22 (-0.29, -0.16)***
Optimism	0.00	-0.05 (-0.10, 0.01)	-0.09 (-0.15, -0.04)**	-0.16 (-0.25, -0.06)**
Purpose in life	0.00	-0.02 (-0.07, 0.03)	-0.10 (-0.16, -0.03)**	-0.23 (-0.32, -0.14)***
Mastery	0.00	-0.04 (-0.11, 0.03)	-0.08 (-0.15, -0.01)*	-0.23 (-0.31, -0.15)***
Health mastery	0.00	-0.01 (-0.09, 0.07)	-0.08 (-0.18, 0.01)	-0.22 (-0.32, -0.13)***
Financial mastery	0.00	0.00 (-0.05, 0.06)	-0.04 (-0.12, 0.04)	-0.16 (-0.23, -0.09)***

© 2022 Nakamura JS et al. *JAMA Network Open.*

eTable 6. (continued)

Outcomes	Aging Satisfaction			
	Quartile 1 (n=1814)	Quartile 2 (n=2051)	Quartile 3 (n=1545)	Quartile 4 (n=1817)
	(Reference)	RR/OR/β (95% CI)	RR/OR/β (95% CI)	RR/OR/β (95% CI)
**Psychological Distress**				
Depression	1.00	1.05 (0.85, 1.29)	1.14 (0.90, 1.44)	1.27 (1.00, 1.62)
Depressive symptoms	0.00	0.04 (-0.04, 0.11)	0.08 (0.01, 0.15)*	0.16 (0.09, 0.23)***
Hopelessness	0.00	0.01 (-0.04, 0.07)	0.09 (0.03, 0.15)**	0.18 (0.12, 0.23)***
Negative affect	0.00	0.04 (-0.02, 0.10)	0.09 (0.03, 0.15)**	0.21 (0.14, 0.27)***
Perceived constraints	0.00	0.05 (-0.01, 0.10)	0.11 (0.05, 0.17)***	0.23 (0.18, 0.29)***
**Social Factors**				
Loneliness	0.00	0.06 (-0.03, 0.15)	0.10 (0.04, 0.17)***	0.20 (0.13, 0.27)***
Not living with a spouse/partner	1.00	1.04 (0.93, 1.16)	1.03 (0.93, 1.15)	1.13 (1.01, 1.26)*
Contact children <1x/week	1.00	0.95 (0.83, 1.08)	0.99 (0.85, 1.15)	0.99 (0.85, 1.14)
Contact other family <1x/week	1.00	1.01 (0.92, 1.11)	1.01 (0.91, 1.13)	1.02 (0.92, 1.13)
Contact friends <1x/week	1.00	0.97 (0.87, 1.08)	0.99 (0.89, 1.11)	0.98 (0.88, 1.10)

Note. Abbreviations: CI, confidence interval; OR, odds ratio; RR, risk ratio.

[a]If the reference value is "1," the effect estimate is OR or RR; if the reference value is "0," the effect estimate is β.

[b]The analytic sample was restricted to those who had participated in the baseline wave (t_1;2012 or 2014) and had a decrease in aging satisfaction between the pre-baseline and baseline wave. Quartile 4 shows the estimates for participants with the largest decreases in aging satisfaction from the pre-baseline to baseline waves, and other quartiles can be interpreted in the same way. Multiple imputation was performed to impute missing data on the exposure, covariates, and outcomes. All models adjusted for sociodemographic characteristics (age, sex, race/ethnicity, marital status, annual household income, total wealth, level of education, employment status, health insurance, geographic region), pre-baseline childhood abuse, pre-baseline religious service attendance, pre-baseline values of the outcome variables (diabetes, hypertension, stroke, cancer, heart disease, lung disease, arthritis, overweight/obesity, physical functioning limitations, cognitive impairment, chronic pain, self-rated health, heavy drinking, current smoking status, physical activity, sleep problems, positive affect, life satisfaction, optimism, purpose in life, mastery, health mastery, financial mastery, depressive symptoms, hopelessness, negative affect, perceived constraints, loneliness, living with spouse/partner, contact children <1x/week, contact other family <1x/week, contact friends <1x/week), personality factors (openness, conscientiousness, extraversion, agreeableness, neuroticism) and the pre-baseline value of the exposure (coded in quartiles). These variables were adjusted for in the wave pre-baseline to the exposure assessment (t_0;2008 or 2010).

[c]An outcome-wide analytic approach was used, and a separate model for each outcome was run. A different type of model was run depending on the nature of the outcome: 1) for each binary outcome with a prevalence of ≥10%, a generalized linear model (with a log link and Poisson distribution) was

© 2022 Nakamura JS et al. *JAMA Network Open.*

used to estimate a RR; 2) for each binary outcome with a prevalence of <10%, a logistic regression model was used to estimate an OR; and 3) for each continuous outcome, a linear regression model was used to estimate a β.

[d]All continuous outcomes were standardized (mean=0; standard deviation=1), and β was the standardized effect size.

*p<0.05 before Bonferroni correction; **p<0.01 before Bonferroni correction; ***p<0.05 after Bonferroni correction (the p-value cutoff for Bonferroni correction is p=0.05/35 outcomes=p<0.001).

© 2022 Nakamura JS et al. *JAMA Network Open.*

eTable 7. Stable Aging Satisfaction and Subsequent Health/Well-Being (Health and Retirement Study [HRS]: N: 4872 to 4881)[a,b,c,d]

**Outcomes**	**Aging Satisfaction**			
	Quartile 1	Quartile 2	Quartile 3	Quartile 4
	(Reference)	RR/OR/β (95% CI)	RR/OR/β (95% CI)	RR/OR/β (95% CI)
**Physical Health**				
All-cause mortality	1.00	0.73 (0.58, 0.92)**	0.69 (0.50, 0.96)*	0.54 (0.38, 0.79)**
Number of chronic conditions	0.00	-0.02 (-0.08, 0.03)	-0.08 (-0.16, 0.01)	-0.14 (-0.22, -0.07)***
Diabetes	1.00	1.02 (0.87, 1.19)	1.04 (0.83, 1.30)	0.99 (0.78, 1.25)
Hypertension	1.00	1.00 (0.90, 1.11)	0.99 (0.87, 1.14)	0.95 (0.82, 1.09)
Stroke	1.00	0.85 (0.65, 1.11)	0.80 (0.54, 1.19)	0.64 (0.42, 0.97)*
Cancer	1.00	1.02 (0.85, 1.22)	0.94 (0.74, 1.21)	0.97 (0.75, 1.24)
Heart disease	1.00	1.02 (0.89, 1.17)	0.93 (0.76, 1.14)	0.77 (0.62, 0.96)*
Lung disease	1.00	1.01 (0.81, 1.27)	0.91 (0.66, 1.25)	0.78 (0.55, 1.12)
Arthritis	1.00	1.00 (0.90, 1.11)	0.96 (0.84, 1.10)	0.91 (0.79, 1.06)
Overweight/obesity	1.00	1.00 (0.90, 1.11)	1.02 (0.89, 1.17)	1.01 (0.87, 1.18)
Physical functioning limitations	1.00	0.82 (0.70, 0.96)*	0.62 (0.50, 0.78)***	0.45 (0.35, 0.58)***
Cognitive impairment	1.00	0.92 (0.77, 1.09)	0.79 (0.62, 1.01)	0.78 (0.60, 1.01)
Chronic pain	1.00	0.88 (0.77, 1.01)	0.82 (0.68, 0.99)*	0.64 (0.52, 0.79)***
Self-rated health	0.00	0.20 (0.11, 0.30)***	0.37 (0.27, 0.46)***	0.50 (0.38, 0.62)***
**Health Behaviors**				
Heavy drinking	1.00	0.95 (0.56, 1.60)	0.89 (0.48, 1.62)	0.79 (0.41, 1.52)
Smoking	1.00	0.86 (0.59, 1.25)	0.80 (0.52, 1.23)	0.86 (0.56, 1.31)
Frequent physical activity	1.00	1.16 (1.01, 1.32)*	1.27 (1.09, 1.49)**	1.29 (1.08, 1.53)**
Sleep problems	1.00	0.92 (0.78, 1.08)	0.85 (0.69, 1.05)	0.78 (0.64, 0.96)*
**Psychological Well-Being**				
Positive affect	0.00	0.14 (0.06, 0.21)***	0.30 (0.22, 0.39)***	0.39 (0.28, 0.51)***
Life satisfaction	0.00	0.20 (0.11, 0.29)***	0.37 (0.25, 0.49)***	0.48 (0.36, 0.59)***
Optimism	0.00	0.05 (-0.04, 0.13)	0.16 (0.05, 0.27)**	0.20 (0.10, 0.31)***
Purpose in life	0.00	0.10 (0.04, 0.17)**	0.24 (0.15, 0.32)***	0.35 (0.26, 0.44)***
Mastery	0.00	0.17 (0.09, 0.25)***	0.27 (0.16, 0.37)***	0.36 (0.24, 0.49)***
Health mastery	0.00	0.20 (0.09, 0.30)**	0.33 (0.18, 0.48)***	0.44 (0.26, 0.62)***
Financial mastery	0.00	0.19 (0.10, 0.28)***	0.25 (0.14, 0.36)***	0.31 (0.19, 0.44)***

© 2022 Nakamura JS et al. *JAMA Network Open.*

eTable 7. (continued)

Outcomes	Aging Satisfaction			
	Quartile 1	Quartile 2	Quartile 3	Quartile 4
	(Reference)	RR/OR/β (95% CI)	RR/OR/β (95% CI)	RR/OR/β (95% CI)
**Psychological Distress**				
Depression	1.00	0.67 (0.52, 0.85)**	0.38 (0.25, 0.58)***	0.24 (0.15, 0.40)***
Depressive symptoms	0.00	-0.28 (-0.36, -0.21)***	-0.41 (-0.51, -0.31)***	-0.41 (-0.51, -0.30)***
Hopelessness	0.00	-0.16 (-0.24, -0.07)***	-0.27 (-0.37, -0.17)***	-0.34 (-0.46, -0.23)***
Negative affect	0.00	-0.17 (-0.26, -0.08)***	-0.25 (-0.35, -0.15)***	-0.37 (-0.48, -0.27)***
Perceived constraints	0.00	-0.16 (-0.25, -0.06)**	-0.32 (-0.43, -0.20)***	-0.43 (-0.55, -0.31)***
**Social Factors**				
Loneliness	0.00	-0.19 (-0.27, -0.11)***	-0.28 (-0.37, -0.18)***	-0.45 (-0.57, -0.32)***
Not living with a spouse/partner	1.00	0.94 (0.83, 1.06)	0.88 (0.74, 1.05)	0.83 (0.69, 1.00)
Contact children <1x/week	1.00	0.98 (0.79, 1.22)	0.91 (0.73, 1.14)	0.91 (0.72, 1.15)
Contact other family <1x/week	1.00	0.98 (0.85, 1.12)	0.97 (0.81, 1.15)	0.96 (0.79, 1.16)
Contact friends <1x/week	1.00	0.93 (0.78, 1.10)	0.93 (0.77, 1.12)	0.93 (0.75, 1.14)

Note. Abbreviations: CI, confidence interval; OR, odds ratio; RR, risk ratio.

[a]If the reference value is "1," the effect estimate is OR or RR; if the reference value is "0," the effect estimate is β.

[b]The analytic sample was restricted to those who had participated in the baseline wave (t_1;2012 or 2014) and remained within the same aging satisfaction quartile between the pre-baseline and baseline wave. Quartile 4 shows the estimates for participants who remained in the highest quartile of aging satisfaction from the pre-baseline to baseline waves, as compared to participants who remained in the lowest quartile (quartile 1) of aging satisfaction from the pre-baseline to baseline waves. Other quartiles can be interpreted in the same way. Multiple imputation was performed to impute missing data on the exposure, covariates, and outcomes. All models adjusted for sociodemographic characteristics (age, sex, race/ethnicity, marital status, annual household income, total wealth, level of education, employment status, health insurance, geographic region), pre-baseline childhood abuse, pre-baseline religious service attendance, pre-baseline values of the outcome variables (diabetes, hypertension, stroke, cancer, heart disease, lung disease, arthritis, overweight/obesity, physical functioning limitations, cognitive impairment, chronic pain, self-rated health, heavy drinking, current smoking status, physical activity, sleep problems, positive affect, life satisfaction, optimism, purpose in life, mastery, health mastery, financial mastery, depressive symptoms, hopelessness, negative affect, perceived constraints, loneliness, living with spouse/partner, contact children <1x/week, contact other family <1x/week, contact friends <1x/week), personality factors (openness, conscientiousness, extraversion, agreeableness, neuroticism) and the pre-baseline value of the exposure (coded in quartiles). These variables were adjusted for in the wave pre-baseline to the exposure assessment (t_0;2008 or 2010).

[c]An outcome-wide analytic approach was used, and a separate model for each outcome was run. A different type of model was run depending on the nature of the outcome: 1) for each binary outcome with a prevalence of ≥10%, a generalized linear model (with a log link and Poisson distribution) was used to estimate a RR; 2) for each binary outcome with a prevalence of <10%, a logistic regression model was used to estimate an OR; and 3) for each continuous outcome, a linear regression model was used to estimate a β.

© 2022 Nakamura JS et al. *JAMA Network Open.*

[d]All continuous outcomes were standardized (mean=0; standard deviation=1), and β was the standardized effect size.
*p<0.05 before Bonferroni correction; **p<0.01 before Bonferroni correction; ***p<0.05 after Bonferroni correction (the p-value cutoff for Bonferroni correction is p=0.05/35 outcomes=p<0.001).

© 2022 Nakamura JS et al. *JAMA Network Open.*
